# A review of the prevalence, trends, and determinants of coexisting forms of malnutrition in neonates, infants, and children

**DOI:** 10.1186/s12889-022-13098-9

**Published:** 2022-05-03

**Authors:** Asif Khaliq, Darren Wraith, Smita Nambiar, Yvette Miller

**Affiliations:** 1 School of Public Health and Social Work, Queensland University of Technology, Brisbane, 4059 Australia; 2 School of Exercise and Nutrition Sciences, Queensland University of Technology, Brisbane, 4059 Australia

**Keywords:** Anthropometry, Child, Coexisting, Malnutrition, Measurement

## Abstract

**Objective:**

*Coexisting Forms of Malnutrition* (CFM) refers to the presence of more than one type of nutritional disorder in an individual. Worldwide, CFM affects more than half of all malnourished children, and compared to standalone forms of malnutrition, CFM is associated with a higher risk of illness and death. This review examined published literature for assessing the prevalence, trends, and determinants of CFM in neonates, infants, and children.

**Methods:**

A review of community-based observational studies was conducted. Seven databases, (CINAHL, Cochrane Library, EMBASE, Medline, PubMed, Scopus, and Web of Science) were used in December-2021 to retrieve literature. Google, Google Scholar and TROVE were used to search for grey literature. Key stakeholders were also contacted for unpublished documents. Studies measuring the prevalence, and/or trends, and/or determinants of CFM presenting in individuals were included. The quality of included studies was assessed using the Joanna Briggs Institute (JBI) critical appraisal tools for prevalence and longitudinal studies.

**Results:**

The search retrieved 14,207 articles, of which 24 were included in this review. The prevalence of CFM varied by geographical area and specific types. In children under 5 years, the coexistence of stunting with overweight/obesity ranged from 0.8% in the United States to over 10% in Ukraine and Syria, while the prevalence of coexisting wasting with stunting ranged from 0.1% in most of the South American countries to 9.2% in Niger. A decrease in CFM prevalence was observed in all countries, except Indonesia. Studies in China and Indonesia showed a positive association between rurality of residence and coexisting stunting with overweight/obesity. Evidence for other risk and protective factors for CFM is too minimal or conflicting to be conclusive.

**Conclusion:**

Evidence regarding the prevalence, determinants and trends for CFM is scarce. Apart from the coexistence of stunting with overweight/obesity, the determinants of other types of CFM are unclear. CFM in any form results in an increased risk of health adversities which can be different from comparable standalone forms, thus, there is an urgent need to explore the determinants and distribution of different types of CFM.

**Supplementary Information:**

The online version contains supplementary material available at 10.1186/s12889-022-13098-9.

## Introduction

**Fig. 1:**
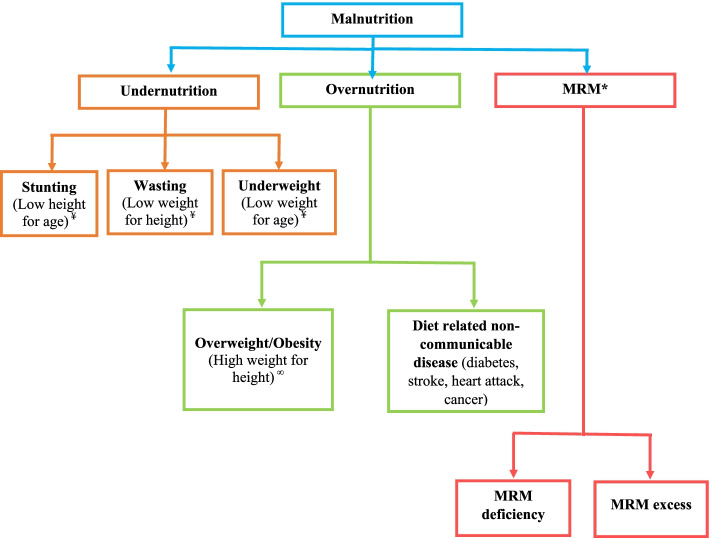
Malnutrition classification and sub-classification. Where, * = Micronutrient Related Malnutrition. ¥ = The z-score is less than − 2.00 S. D or 3rd percentile. ∞ = The z-score is over + 2.00 S. D or 97th percentile

Malnutrition is a global health concern affecting almost every individual, irrespective of age, gender, race, social status, and geographical boundaries [1, 2]. It can be defined as *an imbalance of energy and nutrient intake that may alter the body measurements, compositions and functions* [3, 4]. Thus, malnutrition refers to both undernutrition as well as overnutrition [5]. The World Health Organization (WHO) has classified malnutrition into three broad categories: undernutrition, overnutrition, and Micronutrient-Related Malnutrition (MRM). Stunting, wasting and underweight are three common types of undernutrition, while obesity is related to overnutrition. MRM is further bifurcated into MRM-deficiency and MRM-overload (Fig. 1) [6].

Malnutrition increases the risk of illnesses, treatment costs, hospitalisation, and deaths [7, 8]. Worldwide, 2.4 million or ~ 45% of children below 5 years of age die annually owing to malnutrition [9–11]. The presence of more than one type of nutritional disorder can be referred to as *Coexisting Forms of Malnutrition* (CFM). Children with CFM, such as the coexistence of stunting with wasting, are more vulnerable to death than those with standalone forms of malnutrition [12]. CFM occur due to the simultaneous presence of either multiple anthropometric deficits or MRM or a combination of both, in an individual. Like standalone forms of malnutrition, it can be assessed either by a single method, such as using anthropometric measurements, or multiple methods that involve anthropometry, biochemical and dietary assessment [13–15].

CFM is more complex, challenging to control and is associated with increased health risks compared to standalone forms of malnutrition [16, 17]., McDonald, et al., (2013) found that CFM affects more than half of malnourished children worldwide, and each unit increase in anthropometric deficits proportionally increased the risk of death in children. While children suffering from standalone forms of malnutrition have more than two folds higher risk of death compared to healthy children, this risk increases to more than 10- fold in children suffering from CFM [18]. *The coexistence of stunting with overweight/obesity* and *coexistence of overweight/obesity with micronutrient deficiency* are the two common types of CFM in overweight/obese children. The management and prevention of *coexistence of stunting with overweight/obesity* and/or *coexistence of overweight/obesity with micronutrient deficiency* is more challenging compared to standalone forms of malnutrition because it requires simultaneous prevention and management of overnutrition and undernutrition/micronutrient deficiency [19–21]. Further, evidence for CFM is scarce, as global, national, and regional surveys predominantly measure the prevalence, trends, and determinants of standalone forms of malnutrition, such as stunting, wasting, underweight, overweight/obesity and micronutrient deficiency (Fig. 1).

This scoping review examined the current evidence for existing gaps in the knowledge about the prevalence, trends, and determinants of CFM worldwide in neonates, infants, and children.

## Methodology

### Protocol and registration

The protocol for this review was drafted following PRISMA guidelines and finalised through consultation and review with all authors and an experienced librarian [22]. The PRISMA checklist associated with this scoping review can be found in Supplementary file 1. The protocol was approved by the Human Research Ethics Committee of Queensland University of Technology, Brisbane, Australia (Approval number: 2000000177).

### Eligibility criteria

This study considered official reports from the World Health Organization (WHO), United Nation’s Children Emergency Funds (UNICEF), Centre of Disease Control and Prevention (CDC), Food and Agriculture Organization (FAO), Global Nutrition Report (GNR), Demographic & Health Survey (DHS), Scaling up Nutrition (SUN), and various community-based descriptive and observational epidemiological studies which measured the prevalence, trends, and/or determinants of CFM in children aged between 0 to 12 years irrespective of the sample’s gender, geographical location, and the publication year [23, 24].

Articles that were outside the scope of this review were excluded. These were community-based studies which solely discussed micronutrient deficiencies; studies that described only one type of standalone form of malnutrition; Double Burden of Malnutrition (DBM) at the household level (for example, the coexistence of maternal obesity and paediatric stunting living in the same household); reviews, experimental or intervention trials, institutional-based studies and genomic or molecular level studies; conference proceedings, policy briefs, editorials and book chapters and studies on special populations, such as children with Down’s syndrome, cleft palate, and refugee status due to the different growth trajectories of these children compared to normal children.

### Information sources

Several databases including CINAHL (via EBSCOhost), Cochrane Library, EMBASE, Medline (via EBSCOhost), PubMed, Scopus, and Web of Science were used to identify relevant studies. The literature search was carried out at various time points between 24^th^July, 2019 and 23rd December 2021. The key reports produced by the WHO, UNICEF, CDC, FAO, GNR, DHS, SUN and other relevant bodies were searched using Google, Google scholar and TROVE. In addition, key stakeholders working in epidemiological surveillance, prevention, and control of malnutrition among women and children were contacted for unpublished records and datasets. Altogether, 14,207 studies, including key findings were obtained, published over a 70-year period between 1st-November-1955 to 20th-December-2021. Of these studies, 14,184 were obtained from the aforementioned databases, while the remaining were extracted from the key finding reports of various organizations and governing bodies.

### Search strategy

All members of the research team discussed and developed the search strategy for this review and identified three keywords from the primary research question: *children*, *coexisting forms*, and *malnutrition*. From each keyword, synonyms were searched. In addition, Medical Subject Headings (MeSH) were searched from PubMed and Medline (via EBSCOhost). Keywords, MeSH, and synonyms used for different electronic database searches are presented in Table 1. The Peer Review for Electronic Search Strategies (PRESS) guidelines was consulted to improve the quality of the electronic search process.

**Table 1:** Keywords, MeSH, and Synonyms for identified search terms

Identified Keywords	Synonyms / MeSH
**Child**	Infants, Baby, Toddler, Newborn, Neonate, Paediatric
**Coexisting forms**	Double burden, overlapping, different form
**Malnutrition**	Malnourish, Undernutrition, Overnutrition, Stunting, Wasting, Underweight, Overweight, Obese

### Study selection, data items and data extraction process

**Fig. 2:**
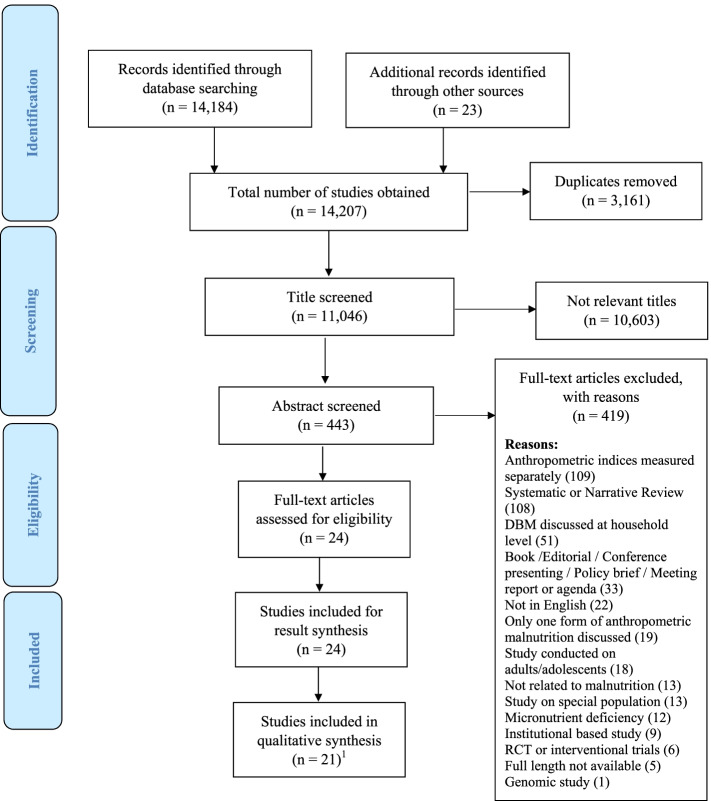
PRISMA Flow Diagram. ^1^ The Global Nutrition Report (GNR) reports were excluded from the quality assessment, because of methodological constraints, i.e., the data collection methods, measurement of exposure, and outcome variable in the GNR report was not described

All studies obtained from different databases were imported to an EndNote library. Within the EndNote library, several functions, such as duplicate removal, title screening, abstract reading, full-text reading, and eligibility determination were performed sequentially by the primary author. Co-authors assisted the primary author to provide clarity through consensus if any studies were unclear. The number of studies included and excluded at each step is presented in Fig. 2.

Studies whose title contained any keyword or synonym related to malnutrition, child, and coexisting forms of malnutrition (Table 1) were considered for abstract and full-text screening. During this phase, the following details were extracted from the articles and tabulated: *study design* (e.g., observational, interventional, review, reports); *study population* (e.g., normal residents or special population); study setting (e.g., community-based or institutional-based); *malnutrition assessment* method (e.g., anthropometry, biochemical test, clinical assessment, dietary assessment)’ *malnutrition assessment level* (e.g., individual, household, community); *malnutrition type* (e.g., standalone or coexisting forms of malnutrition) and *malnutrition factors* (e.g., geographical, socioeconomic, dietary, correlational). These details were used to select studies for inclusion based on the predefined eligibility criteria.

### Summary measures and data synthesis

Study populations, outcomes and statistical methods across the included studies were heterogeneous, so a narrative approach for the synthesis of results was adopted based on Economic and Social Research Council (ESRC) guidelines [25]. The results of all eligible studies were categorised into four groups- *“Definition & Terminology”* (studies that described any phrase, term, or jargon for representing CFM), *“Prevalence”* (studies that described the distribution or prevalence of CFM), *“Trend”* (studies that described changes in the prevalence or burden of CFM with time), or *“Determinants”* (studies that described risk or protective factors for CFM).

### Quality assessment of selected studies

The quality of included studies was assessed using the Joanna Briggs Institute (JBI) critical appraisal tool for prevalence and longitudinal studies. The validity and reliability of the tool have been previously evaluated [26, 27]. The JBI quality assessment scale addresses the reliability and validity of selected studies [28]. Each JBI quality assessment scale measures the quality of studies by four factors: selection; measurement; reporting and attrition. The JBI scale for prevalence studies has nine items, while the JBI quality assessment scale for longitudinal studies has eleven items. Due to the varying number of items in each JBI scale. The JBI assessment system uses four response options for each item: yes, no, unclear and not applicable. The researcher assigned one point for each “Yes”, half point for each “Unclear” or “Not applicable” response and zero points for a “No” answer (Supplementary files 2 and 3).

## Results

### Study characteristics

A total of 14,184 research articles and 23 sources of grey literature were obtained. From those, 24 studies including both research articles and grey literature were included for review (see Table 2). Among the included studies, twenty-one were research studies and three were official reports of Global Nutrition.

**Table 2:** Characteristics of selected studies (*N* = 24)

Publication year	Country	Data	Study design	Study year	Sample size	Sampling method	Study measures	Study population				Data analysis
								Adult age	Indicator	Children age	Indicator	
Florencio, et al., (2001)	Brazil	P	CS	1999	1247	Home to home survey	Prevalence, Determinants	10 to 18 years, Adults over 18 years	BMI	< 10 years	HAZ, WAZ, WHZ	Paired t-test, Multiple regression, variance analysis
Fernald & Neufeld (2007)	Mexico	S	CS	2003	7555	Nil	Prevalence, Determinants	31 ± 9 years	BMI	24 to 72 months	HAZ, BMI*	Multi-nominal logistic analysis
Severi & Moratorio, (2014)	Uruguay	S	CS, LS	2004 to 2011, 2012	4254 children, 3524 women.	Random sampling. Nil	Determinants	13 to 15 years	BMI, Hb-test	6 years and 11 years	HAZ, WAZ, WHZ, BAZ, Hb-level.	Chi-square test.
Kinyoki, et al., (2016)	Somalia	S	CS	2007 to 2010	73,778	Two-stage cluster sampling	Prevalence,	–	–	0 to 59 months	HAZ, WAZ, WHZ	Multivariate spatial technique, Integrated Nested Laplace Approximation (INLA)
Rachmi, et al., (2016)	Indonesia	S	CS	1993, 1997, 2000, 2007	4101	Stratified random sampling	Determinants, Trend.	–	–	24 to 59 months	HAZ, BAZ	Multi-variate analysis model
Zhang, et al., (2016)	China	S	CS	1991, 1993, 1997, 2000, 2004, 2006, 2009	5017	Multistage random-clustered sampling	Prevalence, Determinants, Trend.	–	–	0 to 18 years	HAZ, WAZ, WHZ, BAZ	Multi-nomial logit model
Saaka & Galaa, (2016)	Ghana	S	CS	2014	2720	Stratified cluster sampling	Prevalence, Determinants.	–	–	0 to 59 months	HAZ, WHZ	Moderated hierarchical multiple regression analysis
Mgongo, et al., (2017)	Tanzania	P	CS	2010 to 2011	1870	Multistage sampling	Prevalence	–	–	0 to 24 months	HAZ, WAZ, WHZ, Hb-test	Multivariate logistic regression
Zhang, et al., (2018)	China	S	CS	2016	6570	Multistage sampling	Prevalence	–	–	0 to 59 months	HAZ, WAZ, WHZ, BAZ	Chi-square, Logistic regression
Global Nutrition Report (2018)	Global	S	DDB	Nil	Nil	Nil	Prevalence	Adolescent and Adult	BMI, Hb-test, BP, DM-test, Na-intake	0 to 59 months	HAZ, WHZ	Descriptive
Minh Do, et al., (2018)	Vietnam	P	LS	2013 to 2016	2602	Strategic selection	Trend	–	–	3 to 6 years	HAZ, WHZ	Chi-square
Fongar et al., (2019)	Kenya	S	CS	2016	1058	Two-stage random sampling	Types, Prevalence, Determinants	Adults	BMI	6 to 59 months	HAZ, WAZ, WHZ, MN-testing	t-test
Garenne, et al., (2019)	Senegal	S	LS	1983 to 1984	12,638	Nil	Prevalence, Determinants	–	–	6 to 59 months	HAZ, WAZ, WHZ, HC, MUAC, Skinfolds	Multivariate analysis (linear models & logit-linear model)
Global Nutrition Report (2019)	Global	S	DDB	Nil	Nil	Nil	Prevalence	Adolescent and Adult	BMI, Hb-test, BP, DM-test, Na-intake	0 to 59 months	HAZ, WHZ	Descriptive
Islam & Biswas, (2019)	Bangladesh	S	CS	2014	6965	Two-stage stratified sampling	Types, Prevalence, Determinants	–	–	0 to 59 months	HAZ, WAZ, WHZ	Multivariable logistic regression
Varghese & Stein (2019)	India	S	CS	2015 to 2016	145,653	Stratified, two-stage probability sampling	Types, Prevalence	Women (15 to 49 years)	BMI, Hb-level	6 to 59 months	HAZ, WAZ, Hb-level	Chi-square, correlation, and linear regression
Yasmin, et al., (2019)	Indonesia	S	CS	2010	8599	Two-stage sampling	Prevalence, Determinants	–	–	6 to 12 years	HAZ, BAZ	Chi-square test, Multivariate logistic regression
Ferreira, (2020)	Brazil	S	CS	1992 and 2015	1229 and 987	Multistage Probability sampling	Types, Determinants, Trend	–	–	0 to 59 months	HAZ, WAZ, WHZ,	Chi-square test,
Benedict, et al., (2020)	Thailand	S	CS	2015 to 2016	12,313	Multistage stratified cluster sampling	Prevalence, Determinants	–	–	0 to 59 months	HAZ, WHZ, BAZ	Chi-square test, Multiple Poisson regression
Global Nutrition Report (2021)	Global	S	DDB	Nil	Nil	Nil	Prevalence	Adolescent and Adult	BMI, Hb-test, BP, DM-test, Na-intake	0 to 59 months	HAZ, WHZ	Descriptive
Farah, et al., (2021)	Ethiopia	S	CS	2015	8714	Multistage stratified cluster sampling	Prevalence, Determinants	–	–	0 to 59 months	HAZ, BAZ	Hierarchical logistic regression
Zhang, et al., (2021)	China	P	CS	2016	110,491	Multistage stratified cluster sampling	Prevalenc	–	–	1 to 83 months	HAZ WAZ BAZ	Chi-square, one sampled Wilcoxon-sign ranked test, Multivariate logistic regression
Roba, et al., (2021)	Ethiopia	S	CS	2019	1200	Simple random sampling	Prevalence, Determinants	ND	BMI	0 to 59 months	HAZ, WAZ, WHZ, MUAC	Multivariate binary logistic regression
Khaliq, et al., (2021)	Pakistan	S	CS	2012–2013, 2017–2018	6168	Multistage stratified cluster sampling	Prevalence, Trends, Determinants	–	–	0 to 59 months	HAZ, WAZ, WHZ	Multivariate logistic regression

*BAZ* BMI for age Z-score, *BMI* Body Mass Index, BMI* Body Mass Index for age percentile, *BP-test* Blood pressure measurement, *CS* Cross-sectional, *DDB* Different Databases, *DM –test* Diabetes testing, *HC* Head Circumference, *HAZ* Height for Age Z-score, *Hb-test* Haemoglobin test for anaemia, *LS* Longitudinal study, *ME* Modelled Estimates, *MN-testing* Micronutrient testing, *MUAC* Measuring Upper Arm Circumference, *Na-intake* Sodium intake, *ND* Not defined, *Nil* No information obtained from the review article, *P* Primary data source, *S* Secondary data source, *WAZ* Weight for Age Z-score, *WHZ* Weight for Height Z-score, ¥ The GNR reports reported prevalence of coexisting forms of malnutrition, but due to yearly reporting it was considered as a trend

**Fig. 3:**
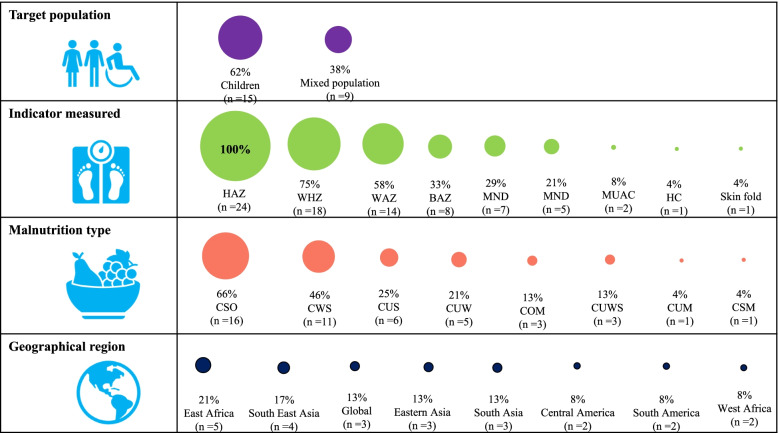
Characteristics of included studies. HAZ = Height for Age z-scores, WHZ = Weight for Height z-scores, WAZ = Weight for Age z-scores, BMI = Body Mass Index, BAZ = Body Mass Index for Age z-scores, MND = Micronutrient deficiency, HC = Head circumference, MUAC = Measuring upper arm circumference, CSO = Coexistence of stunting with overweight/obesity, CWS = Coexistence of wasting with stunting, CUS = Coexistence of underweight with stunting, CUW = Coexistence of underweight with wasting, COM = Coexistence of overweight/obesity with micronutrient deficiency, CUWS = Coexistence of underweight with wasting and stunting, CUM = Coexistence of underweight with micronutrient deficiency, CSM = Coexistence of stunting with micronutrient deficiency

**Fig. 4:**
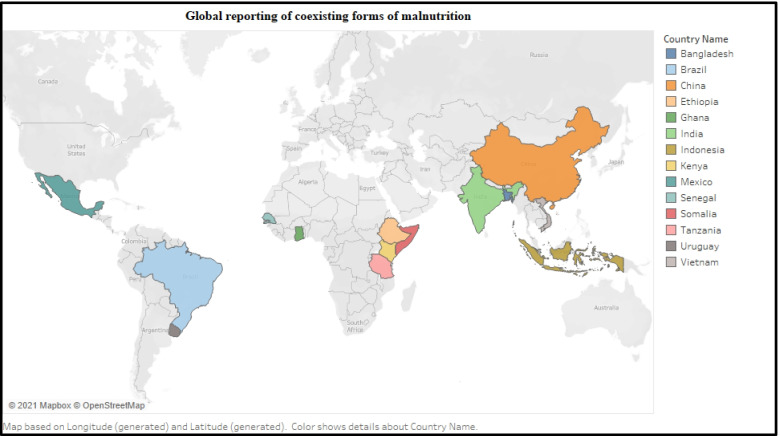
Global reporting of coexisting forms of malnutrition

The outcome variables were anthropometric indices in all selected studies, while the exposure variables included sociodemographic, socioeconomic, geographic, dietary, illness and health-related factors. Among the 24 studies, fifteen studies presented CFM specifically in children, while the remaining 9 studies examined CFM in children, adolescents and adults. Together, the included studies reported CFM in the following countries: Bangladesh, Brazil, China, Ethiopia, Ghana, India, Kenya, Indonesia, Mexico, Pakistan, Senegal, Somalia, Tanzania, Thailand, Uruguay, and Vietnam. Further characteristics are outlined in Figs. 3 and Fig. 4.

### Definitions and terminologies for representing coexisting forms of malnutrition

Several terminologies were used to describe the presence of more than one form of malnutrition. These include concurrent existence of malnutrition [12, 29–32], coexisting forms of malnutrition [33, 34], short and plump syndrome [35], decompensated chronic undernutrition [36] paradox [37] and dual/double burden of malnutrition (DBM) [35, 38, 39]. The term DBM was used to describe individuals who were simultaneously suffering from undernutrition and overnutrition, for example, the coexistence of stunting with overweight/obesity or overnutrition with micronutrient deficiencies (i.e., the coexistence of overweight/obesity with anaemia) [20, 40].

The Global Nutrition Report identified two different types of CFM in children (specifically, with stunting): coexistence of stunting with overweight/obesity, and coexistence of wasting with stunting [41–43], and these types of CFM were also described by Ferreira (2020) [36]. Four other studies identified different presentations for CFM. Fongar, et al., (2019) identified three different presentations of CFM (specifically, with obesity) at an individual level: (i) obesity with micronutrient deficiency in adults, (ii) obesity with micronutrient deficiency in children and (iii) stunting with overweight/obesity. These identified combinations represent contrasting forms of malnutrition and are also known as DBM [35]. Varghese, et al. (2019), described five different presentations for CFM in children: (i) anaemia with overweight (ii) anaemia with underweight (iii) anaemia with stunting (iv) stunting with overweight and (v) stunting with underweight. Varghese, et al. (2019), also identified anaemia with underweight and anaemia with overweight in women [38]. Islam & Biswas described three different types of coexisting forms of undernutrition (specifically, with underweight): (i) underweight with wasting, (ii) underweight with stunting, and (iii) underweight with wasting and with stunting [44]. However, Khaliq, et al., (2021) presented four different types of CFM: (i) coexistence of underweight with wasting, (ii) coexistence of underweight with stunting, (iii) coexistence of underweight with both wasting and stunting, and (iv) coexistence of stunting with overweight/obesity [45].

### Prevalence of coexisting forms of malnutrition

Twenty studies presented the prevalence of CFM. Of these, eleven studies discussed more than one type of CFM.

Most studies (*n* = 14) examined the coexistence of stunting with overweight/obesity, followed by wasting with stunting (*n* = 9); coexistence of underweight with stunting (*n* = 7) and underweight with wasting (*n* = 5). The coexistence of underweight with both wasting and stunting was reported by three studies [44–46]. Two studies presented the burden of coexistence of micronutrient deficiency with undernutrition (stunting or underweight) or with overweight/obesity [35, 38] (Table 3).

**Table 3:** Prevalence of coexisting forms of malnutrition worldwide (*N* = 20)

Author name (Year)	Country	The burden of various types of coexisting forms of malnutrition in neonates, infants, and children					
		Coexistence of undernutrition				Contrasting forms of malnutrition	Coexistence with MRM
		CWS (***n*** = 9)	CUS (***n*** = 7)	CUW (***n*** = 5)	CUWS (***n*** = 3)	CSO (*n* = 14)	COM (***n*** = 2)
**Global Prevalence of CFM (*n*** **= 3)**							
Global Nutrition Report (2018)	Global	5%^AS^ 2.9%^AF^ 0.2%^E^				0.8% ^USA^ 2.5% ^E + AF^	–
Global Nutrition Report (2019)	Global	3.6%	–	–	–	1.9%	–
Global Nutrition Report (2021)	Global	Figure-5				Figure-5	
**CFM prevalence in Asia (*n*** **= 9)**							
Zhang, et al., (2016)	China	–	–	–	–	5%	–
Mgongo, et al., (2017)	Tanzania	12%	33%	21%	12%	–	–
Zhang, et al., (2018)	China	–	–	–	–	18%	–
Islam & Biswas, (2019)	Bangladesh	–	18%	5.5%	5.7%	–	–
Varghese & Stein (2019)	India	–	3.3% (IQR: 2.1 to 5.4) ^α^			0.7% (IQR: 0.4 to 1.2) ^α^	A + OW =0.8% (IQR: 0.5 to 1.3) ^α^ A+ UW = 11.3% (IQR: 8.5 to 13.8) ^α^ A + S = 15.9% (IQR: 12.9 to 20.2) ^α^
Yasmin, et al., (2019)	Indonesia	–	–	–	–	7.5%	–
Benedict, et al., (2020)	Thailand	–	–	–	–	1.6%	
Zhang, et al., (2021)	China	0.2%	1.7%	2.3%	–	0.4%	–
Khaliq, et al., (2021)	Pakistan	–	17.2% ^¥^ 14.3%^¥¥^	2.9% ^¥^ 3.1%^¥¥^	4.4% ^¥^ 2.7%^¥¥^	6.1% ^¥^ 1.4%^¥¥^	–
**CFM prevalence in Africa (*n*** **= 6)**							
Kinyoki, et al., (2016)	Somalia	9%	29%	20%			
Saaka & Galaa (2016)	Ghana	1.4%					
Fongar et al., (2019)	Kenya	–	–	–	–	1%	19%**
Garanne, et al., (2019)	Senegal	6.%	–	–	–	–	–
Farah, et al., (2021)	Ethiopia	–	–	–	–	2% (95% CI: 1.6 to 2.5)	–
Roba, et al., (2021)	Ethiopia	5.8%	–	–	–	–	–
**CFM prevalence in South and Central America (*n*** **= 2)**							
Florencio, et al., (2001)	Brazil	–	8.7% 2.7%*	–	–	0% 30%*	–
Fernald & Neufeld (2007)	Mexico	–	–	–	–	5 to 10%	–

Where, *AS* Asia, *AF* Africa, *E* Europe, *USA* United States of America, *E-AF* Europe and Africa, * = Adolescents, ** = Adult, *CWS* Coexistence of wasting with stunting, *A + OW* Coexistence of Anaemia with Overweight/Obesity, *A + UW* Coexistence of Anaemia with underweight, *A + S* Coexistence of Anaemia with Stunting, α Median prevalence of malnutrition, *IQR* Interquartile range, ¥ = the Survey year 2012–2013, ¥¥ = Survey year 2017–2018, *CUS* Coexistence of underweight with stunting, *CUW* Coexistence of underweight with wasting, *CUWS* Coexistence of underweight with stunting and with wasting, *CSO* Coexistence of overweight/obesity with stunting, *COM* Coexistence of overweight/obesity with micronutrient related malnutrition

The prevalence of CFM varied according to the geographical area and target population. Globally, around 1.7% of children below 5 years of age were affected with the coexistence of stunting with overweight/obesity [42]. The prevalence of coexistence of stunting with overweight/obesity among children under 5 years old was 2% in Ethiopia [30], 1% in India [38], 7.5% in Indonesia [31], 1% in Kenya [35]; between 5 and 10% in Mexico [29]; 1.4–6.1% in Pakistan [45], 1.6% in Thailand [47], 2–3% in Uruguay [39], and 0.4–18% in China [37, 48, 49].

According to the 2019 Global Nutrition Report, the global prevalence of coexistence of wasting with stunting among children below 5 years of age was 3.5% [42]. The coexistence of wasting with stunting was most prevalent in Asian countries (5%), followed by African countries (2.9%), and lower again in European countries, at 2% [41]. Most of the studies conducted in Asia, Africa, and South America reported that the prevalence of coexistence of wasting with stunting was between 5 and 12% in children under the age of 5 years [12, 46, 50–53]. However, two studies conducted in China and Ghana reported a lower prevalence of coexistence of wasting with stunting in children 0.2 and 1.4%, respectively [49, 54].

In children under 5 years of age, the prevalence of coexistence of underweight with stunting was 18% in Bangladesh [44], 9% in Brazil [52], 1.7% in China [49], 14.3–17.2% in Pakistan [45], 29% in Somalia [50] and 33% in Tanzania [46]. Coexistence of underweight with wasting had a reported prevalence of 2.3% in China [49], 6% in Bangladesh [44], 2.9–3.1% in Pakistan [45], 20% in Somalia [50] and 21% in Tanzania [46]. The coexistence of underweight with both wasting and stunting was 5.7% in Bangladesh [44], 2.7–4.4% in Pakistan [45], and 12% in Tanzania [46] (Table 3).

Two studies described the coexistence of micronutrient deficiencies with either undernutrition (stunting, or wasting, or underweight) or overnutrition (overweight/obesity). Iron Deficiency Anaemia was discussed as micronutrient deficiency in both studies [35, 38]. Fongar, et al., 2019 also assessed micronutrient deficiencies of zinc and vitamin-A, in addition to iron [35]. The burden of coexistence of overweight/obesity with micronutrient deficiency in India was 0.8% [38], while in Kenya, the prevalence of coexistence of overweight/obesity with micronutrient deficiency reported was 19% [35] (Table 3).

**Fig. 5:**
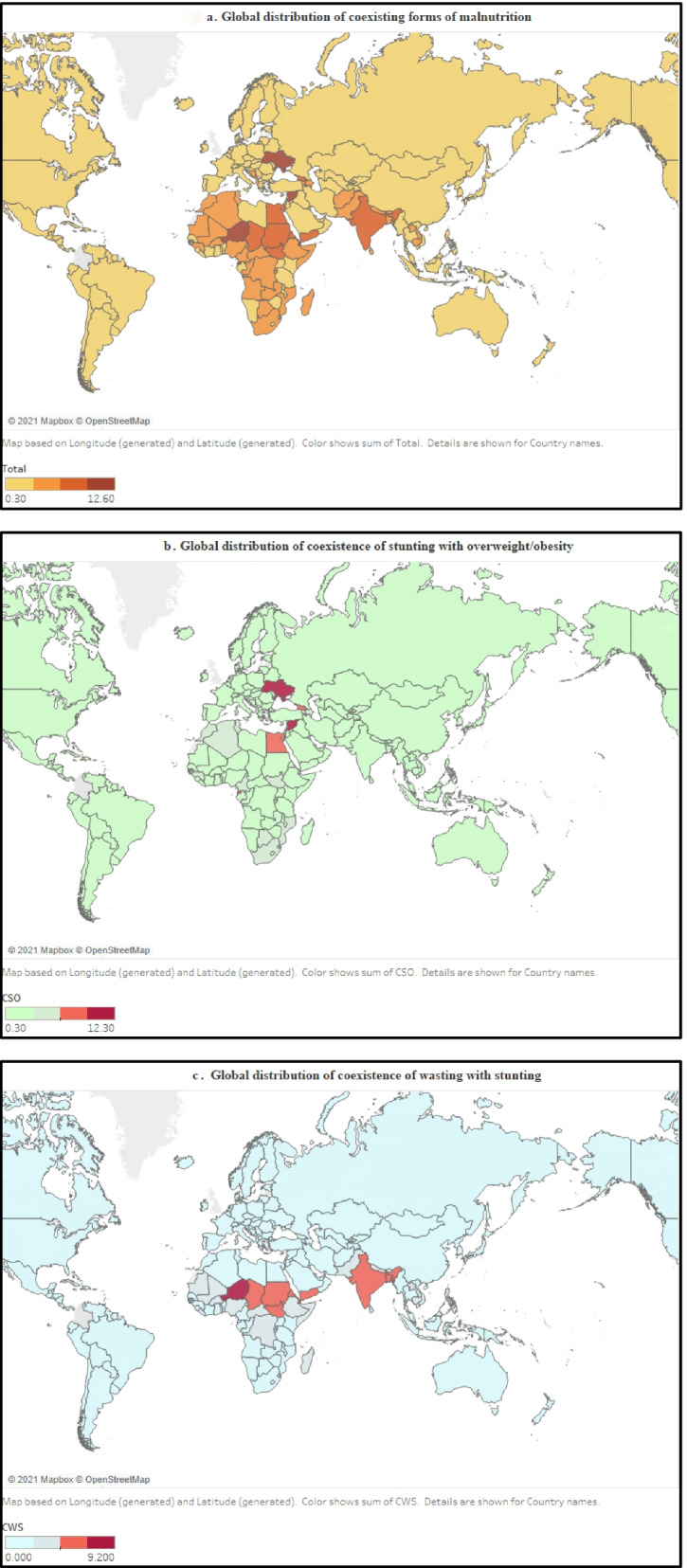
**a** Global prevalence of coexisting forms of malnutrition (CFM)*. **b** Global prevalence of coexistence of stunting with overweight/obesity. **c** Global prevalence of coexistence of wasting with stunting. Where * shows the CFM is the sum of coexistence of stunting with overweight/obesity and coexistence of wasting with stunting in children below 5 years. The detail regarding country-specific prevalence for each form of CFM, including coexistence of stunting with overweight/obesity and coexistence of wasting with stunting can be accessed from Supplementary file 4

The 2021 Global Nutrition Report only presented the country-wise prevalence of two major types of CFM: the coexistence of stunting with overweight/obesity and the coexistence of wasting with stunting. The highest prevalence of CFM was reported for Ukraine, Syria, Equatorial Guinea, and Djibouti. Most countries (*n* = 76 of 110) reported CFM prevalence between 1 and 4.9%. Ukraine and Syria had the highest reported prevalence of coexistence of stunting with overweight/obesity in children (12.3 and 11.1%, respectively). However, the highest prevalence of coexistence of wasting with stunting (9.2%) was observed in Niger, although the prevalence of coexistence of wasting with stunting over 5% was also reported for Bangladesh, Chad, Djibouti, Eritrea, India, South Sudan, Sudan, Timor-Leste, and Yemen. The geographical distribution of CFM and its two major types (coexistence of stunting with overweight/obesity, and coexistence of wasting with stunting) is represented in Figs. 5a-c. The exact statistics regarding the prevalence of CFM and its specific types of CFM can be accessed from Supplementary file 4.

### Trends in coexisting forms of malnutrition

Trends in the prevalence of CFM over time were reported in five studies [32, 33, 36, 37, 45]. Four studies reported trends for the coexistence of stunting with overweight/obesity, and one reported the trend for coexistence of wasting with stunting, in Brazil. The trends of coexistence of underweight with wasting, the coexistence of underweight with stunting, and coexistence of underweight with both wasting and stunting was reported by one study [45]. Coexistence of stunting with overweight/obesity in Indonesia increased from 6.4% (95% CI: 5 to 8.2) in 1993 to 7.2% (95% CI: 6 to 8.8) in 2007 in children aged between 2 to 5 years of age [33]. However, other studies conducted in Brazil, China, Pakistan, and Vietnam reported a decline in different forms of CFM. In Brazil, the coexistence of wasting with stunting in children under 5 years of age decreased from 0.5% in 1992 to 0% in 2015 [36]. In rural areas of China, the coexistence of stunting with overweight/obesity among children and adolescents decreased from 26% in 1991 to 6% in 2009 [37]. Pakistan showed a significant decline in coexistence of stunting with overweight/obesity in 2017–2018, compared to the former survey of 2012–2013 [45]. In Vietnam, the prevalence of coexistence of stunting with overweight/obesity decreased from 2.7% in 2013 to 1.4% in 2016 in children aged over 3 years [32].

#### Contributing factors of coexisting forms of malnutrition

The contributing factors of CFM were reported in 13 studies (summarised in Table 4), including:

**Table 4:** Contributing factors for coexisting forms of malnutrition (*N* = 15)

Author name (Year)	Country	Malnutrition type	Contributing factors for coexisting forms of malnutrition															
			Age	Sex	Birth size & birthweight	Birth interval and birth order	Food and diet	Health and disease status	Health insurance	Antenatal consultations	Parent’s education	Maternal occupation	Parental obesity & short stature	Maternal age	Socioeconomic stratus	Family size	Water, sanitation, and toilet	Region
Florencio, et al., (2001)	Brazil	CUW	**✓**															
Fernald & Neufeld (2007)	Mexico	CSO	✓	✓														
Severi & Moratorio, (2014)	Uruguay	CSO	**✓**								**✓**	**✓**	**✓**	**✓**	**✓**			
Rachmi, et al., (2016)	Indonesia	CSO	**✓**				**✓**						**✓**					**✓**
Saaka & Galaa (2016)	Ghana	CWS	**✓**	**✓**														
Zhang, et al., (2016)	China	CSO	**✓**	**✓**					**✓**		**✓**				**✓**			**✓**
Garenne, et al., (2018)	Senegal	CWS	**✓**	**✓**				**✓**										
Fongar, et al., (2019)	Kenya	CSO	**✓**	**✓**							**✓**				**✓**			
Islam & Biswas, (2019)	Bangladesh	CFU^a^	**✓**	**✓**	**✓**	**✓**		**✓**		**✓**	**✓**	**✓**	**✓**	**✓**		**✓**		**✓**
Yasmin, et al., (2019)	Indonesia	CSO	**✓**	**✓**			**✓**				**✓**		**✓**	**✓**	**✓**	**✓**		**✓**
Ferreira, (2020)	Brazil	CWS	**✓**	**✓**														
Benedict, et al., (2020)	Thailand	CSO	**✓**	**✓**							**✓**				**✓**	**✓**		**✓**
Farah, et al., (2021)	Ethiopia	CSO	**✓**	**✓**	**✓**		**✓**	**✓**			**✓**				**✓**		**✓**	**✓**
Roba, et al., (2021)	Ethiopia	CWS	**✓**	**✓**				**✓**									**✓**	
Khaliq, et al., (2021)	Pakistan	CUW CUS CUWS CSO	**✓**	**✓**							**✓**	**✓**			**✓**	**✓**		**✓**

*CWS* Coexistence of wasting with stunting, *CFU* Coexisting forms of undernutrition, *CSO* Coexistence of overweight/obesity with stunting, *COM* Coexistence of overweight/obesity with micronutrient related malnutrition

^a^Islam & Biswas (2019) assessed the determinants of coexistence of underweight with wasting (CUW), the coexistence of underweight with stunting (CUS), and coexistence of underweight with wasting and stunting (CUWS), jointly. Thus, they assessed the determinants of coexisting forms of undernutrition, i.e., CFU

##### Age

A study conducted in Brazil showed a 6% prevalence of coexistence of wasting with stunting in children aged between 0 and 24 months, while in older children aged over 24 months no cases of coexistence of wasting with stunting were reported [36]. Garenne, et al., reported that children aged between 12 to 23.99 months had the highest burden of coexistence of wasting with stunting among children under 5 years [12]. Saaka and Galaa (2016) reported that in children under 5 years of age, a high prevalence of coexistence of wasting with stunting was observed in children aged between 6 to 35 months, compared to children aged between 36 to 59 months [54]. The coexistence of wasting with stunting was more common among children below 10 years than for adolescents and adults [52]. In contrast, high odds of coexistence of underweight with stunting, and coexistence of underweight with both wasting and stunting were reported by Khaliq, et al., (2021) in children aged over 1 year [45]. Islam & Biswas, and Roba, et al., (2021) in their study did not find a significant association between CFM and child age [44, 53]. The problems relating to undernutrition appear to diminish with increasing age, while the probability of coexistence of stunting with overweight/obesity or coexistence of overweight/obesity with micronutrient deficiency rises [30, 33, 39, 52]. In contrast, Zhang, et al., (2016) reported that every year of increasing age significantly reduced the odds of coexistence of stunting with overweight/obesity by 0.74 (95% CI: 0.71 to 0.77) [37]. Similarly, Khaliq, et al., (2021) reported lower odds of coexistence of stunting with overweight/obesity in children older than one-year [45]. In another study, the odds of coexistence of stunting with overweight/obesity in children aged three to 5 years was significantly higher (OR: 1.4, 95% CI: 1.0 to 1.8), compared to children aged under 2 years [29]. Benedict, et al., (2020) identified that children aged between 24 to 47 months had over three-to-four fold higher odds of coexistence of stunting with overweight/obesity, compared with younger infants [47]. In another study, the odds of coexistence of stunting with overweight/obesity was over four times higher in children aged below 1 year, compared with children aged 48 to 59 months [30]. The prevalence of coexistence of stunting with obesity in young school-aged children (6 to 9 years) and older children aged (10 to 12 years by Yasmin, et al., (2019) was 8.1, and 6.9%, respectively [31]. Overall, the coexistence of wasting with stunting is more common among young children, but conflicting associations between the coexistence of stunting with overweight/obesity and child age was observed. Hence, the available evidence is not sufficient to ascertain a vulnerable age group for the coexistence of stunting with overweight/obesity.

##### Gender

Both boys and girls were susceptible to CFM. Among children under 5 years of age, many studies reported a higher prevalence of CFM in boys compared with girls. Studies conducted in China, Ethiopia, Kenya, and Thailand reported a higher prevalence of coexistence of stunting with overweight/obesity and coexistence of wasting with stunting in boys [35, 37, 47, 53]. The prevalence of coexistence of stunting with overweight/obesity among the male and female children of Indonesia was 1.15 and 0.84%, respectively [31]. However, several other studies conducted in Brazil, China, Ethiopia, Ghana, and Pakistan found no association of gender with the coexistence of stunting with overweight/obesity or coexistence of wasting with stunting in children [30, 36, 37, 45, 54].

### Birth size and birthweight

One study in Ethiopia found no association between the coexistence of stunting with overweight/obesity and birth size [30]. The Bangladesh Demographic and Health Survey (BDHS) of 2011 indicated that the odds of coexisting forms of undernutrition in average or large-sized children was almost two-times higher than the smaller size children [44].

### Birth interval and birth order

A study conducted in Bangladesh showed no association between coexisting forms of undernutrition and birth interval but reported a significant association with birth order. Coexistence of underweight with stunting, the coexistence of underweight with wasting, and coexistence of underweight with wasting and stunting decreased as the birth order increased [44].

### Food accessibility and diet

In one study in Indonesia, prolonged weaning over 6 months was associated with the coexistence of wasting with stunting [33]. Micronutrient supplementation with iron showed no association with the coexistence of stunting with overweight/obesity [30]. Yasmin, et al., (2019) identified that energy inadequacy significantly predicted the coexistence of stunting with overweight/obesity in children, while protein adequacy levels and adequate consumption of carbohydrates, proteins and fat was not associated with the coexistence of stunting with overweight/obesity [31]. However, the available evidence is limited, and further research is needed to explore the influence of different foods to identify their role in the prevention and management of various types of CFM.

#### Health and disease status

Islam & Biswas (2020) reported no association between CFM and childhood illnesses, such as diarrhoea, acute respiratory tract infection, and fever. Compared to vaccinated children, the non-vaccinated children have almost two-folds higher odds of coexistence of underweight with stunting, the coexistence of underweight with wasting, or coexistence of underweight with wasting and stunting [44]. Roba, et al., (2021) reported a protective effect of the absence of illnesses for the coexistence of wasting with stunting in children, compared with children with a previous history of preventable childhood illness [53]. In children under 5 years of age in Ethiopia, the odds of coexistence of stunting with overweight/obesity was two-folds higher in healthy children compared to infected children. Similarly, children who had not received deworming tablets in the last 6 months also exhibits over two-folds higher odds of coexistence of stunting with overweight/obesity than those who had received deworming tablets [30].

### Health insurance

Zhang, et al., (2016) found no association between health insurance and the coexistence of stunting with overweight/obesity [37].

### Antenatal consultation visit

Islam & Biswas (2020) found no significant association between antenatal consultation visits and CFM [44].

#### Education

Zhang, et al., (2016) reported that maternal education provided a protective effect against the coexistence of stunting with overweight/obesity [37]. Similarly, Fernald & Neufeld (2006) reported that maternal education over the primary level significantly decreased the odds of coexistence of stunting with overweight/obesity in children by 0.59 (95% CI: 0.42 to 0.82) [29]. However, a study conducted in Thailand showed around two-fold higher odds of coexistence of stunting with overweight/obesity in children with mothers with secondary level education, compared with highly educated mothers (higher education) [47]. Studies conducted in Ethiopia, Indonesia, Kenya, and Pakistan did not show a significant relationship of caregiver education with the coexistence of stunting with overweight/obesity [30, 33, 35, 45]. Similarly, studies conducted in Bangladesh and Pakistan showed no association between parent’s education and coexistence of underweight with stunting, the coexistence of underweight with wasting, or coexistence of underweight with wasting and stunting both [44, 45]. An Indonesian study also found no association between maternal education and coexistence of stunting with overweight/obesity, but paternal education was negatively associated with the coexistence of stunting with overweight/obesity [31].

### Maternal occupation

Secondary analysis of data from the BDHS 2011 showed no association of maternal working status with the coexistence of underweight with stunting, the coexistence of underweight with wasting, or coexistence of underweight with wasting and stunting in children under 5 years of age [44]. However, secondary data analysis of Pakistan Demographic and Health Survey (PDHS) 2012–2013 and 2017–2018 showed a protective effect of maternal employment for the coexistence of underweight with wasting, and coexistence of stunting with overweight/obesity [45].

### Parental obesity and short stature

Some studies reported no relationship between parental obesity and coexistence of stunting with overweight/obesity in children [29, 33]. Yasmin, et al. (2019) found a significant relationship between paternal BMI, but not maternal BMI, and the coexistence of stunting with overweight/obesity [31]. Islam and Biswas found no significant relationship between maternal BMI and other types of CFM (coexistence of underweight with stunting, the coexistence of underweight with wasting, and coexistence of underweight with wasting and stunting both) [44]. Two studies from Mexico and Indonesia reported that short maternal stature increased the odds of coexistence of stunting with overweight/obesity in children to 1.66 (95% CI: 1.22 to 2.58), compared with children of normal maternal stature [29, 33]^.^

### Maternal age

A study conducted in Mexico by Fernald & Neufeld reported that children of mothers aged below 18 years were at increased risk of coexistence of stunting with overweight/obesity [29]. Yasmin, et al. (2019) found that the coexistence of stunting with overweight/obesity in children under 5 years of age in Indonesia was not associated with either maternal or paternal age [31]. Islam & Biswas (2020) reported an increase in the odds of coexisting forms of undernutrition to 1.63 (95% CI: 1.03 to 2.59) among children born to mothers under 20 years of age, compared to mothers aged over 20 years [44].

### Socioeconomic status

A complicated relationship of socioeconomic status (SES) with different forms of malnutrition was evident. Islam and Biswas found that low SES was significantly associated with the coexistence of underweight with stunting, the coexistence of underweight with wasting, or coexistence of underweight with wasting and stunting [44]. Similarly, Khaliq, et al., (2021) reported lower odds of coexistence of underweight with stunting, the coexistence of underweight with wasting, or coexistence of underweight with wasting and stunting in children of richer/richest SES, compared to children of poorest SES [45]. However, studies conducted in China, Ethiopia, India, Kenya, and Thailand showed no association of any forms of CFM with SES [30, 35, 37, 38, 47]. Further research is needed to demonstrate the relationship of CFM with SES.

#### Family size

In one study, the odds of developing coexistence of stunting with overweight/obesity among Mexican children increased significantly by 1.16 (95% CI: 1.09 to 1.22) for each additional family member [29]. In contrast, Benedict, et al., (2020) reported around two folds higher odds of coexistence of stunting with overweight/obesity in those households having two or more children [47]. However, an Indonesian study reported no relationship of family size with the coexistence of stunting with overweight/obesity [31]. Similarly, a study conducted in Pakistan did not show any relationship of family size with any forms of CFM [45].

#### Water source and toilet facility

Two studies conducted in Ethiopia assessed the relationship of CFM with the source of drinking water. Roba, et al., (2021) reported over three times higher odds of coexistence of wasting with stunting in children who had a non-chlorinated water supply, compared to children having chlorinated water supply [53]. Farah, et al., (2021) did not observe a significant relationship of type of water source with the coexistence of stunting with overweight/obesity. However, Farah, et al., (2021) observed a significant relationship between toilet type and coexistence of stunting with overweight/obesity. The presence of unimproved toilet facilities approximately doubled the odds of coexistence of stunting with overweight/obesity compared with children with improved toilet facilities in their households [30].

#### Urbanization

High levels of urbanisation have been shown to reduce the risk of all types of undernutrition disorders, including the coexistence of stunting with overweight/obesity. In China, an increase in urbanization significantly decreased the risk of coexistence of stunting with overweight/obesity by 43% [37], whereas studies conducted in Pakistan and Thailand demonstrated around two-fold higher odds of coexistence of stunting with overweight/obesity in children of urban residence [45, 47]. In contrast, findings from two studies conducted in Indonesia showed significantly higher odds of coexistence of stunting with overweight/obesity in children of rural areas compared with children of urban areas [31, 33]. In contrast, there was no significant relationship between the coexistence of stunting with overweight/obesity and area of residence in Ethiopia [30]. Islam and Biswas reported that urbanization increased the likelihood of coexisting forms of undernutrition in children of Bangladesh [44].

### Quality assessment of included studies

**Fig. 6:**
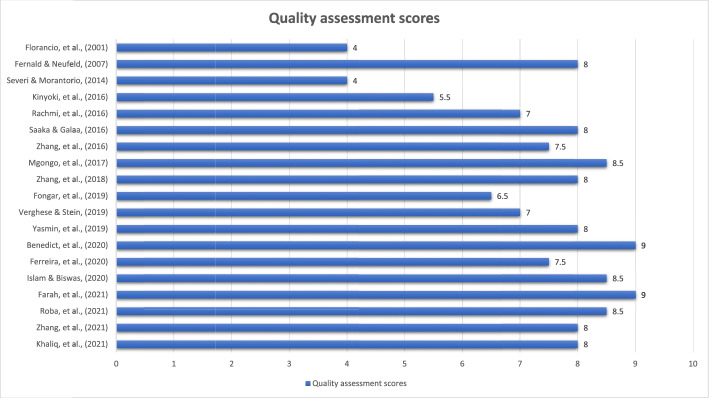
Quality assessment score of each prevalence study

Out of twenty-four studies, twenty-one studies were selected for quality assessment, while all the global nutrition reports (Grey literature) were excluded from quality assessment because of a lack of methodological information (i.e., the data collection methods, measurement of exposure, and outcome variable in the GNR report was not described) [41–43]. Among the twenty-one studies selected for quality assessment, nineteen were prevalence studies. Amongst the prevalence studies, five scored 8.5 or 9 out of 9, and 12 studies scored between 5.5 to 8. Two prevalence studies scored less than 4.5 out of 9 [39, 52]. The two longitudinal studies selected for quality assessment scored 5.5 and 7.5 out of 11 (Fig. 6).

Studies that adequately defined sampling frame, sample population, sample size, study design and the data collected by probability sampling method in a defined study setting were defined as having an appropriate selection criterion. There were 16 studies, which had appropriately defined selection criteria [29–31, 33, 35–38, 44, 46–49, 53, 54]. Eighteen studies measured the exposure and outcome variable appropriately [29–33, 35–38, 44, 46–49, 52–54]. In this review, appropriate measure refers to the measurement of exposure and outcome variables using a valid and reliable method. Studies in which anthropometric measurement was performed by trained and professional data collectors showed good measurement standards. For assessing the nourishment status of adults, the BMI classification proposed by WHO was used, while for children, tools such as National Centre for Health Statistics (NCHS)) and WHO child growth standards 2006 were used. In this review, there were twenty studies published after 2006, of which seventeen used WHO child growth for assessing the outcome. The remaining studies published after 2006 used NCHS and Chinese growth references for calculating child anthropometry (Table 5).

**Table 5:** Anthropometry assessment method

	National centre for health statistics (*n* = 3)	WHO Child growth standard, 2006 (*n* = 17)	Chinese growth reference (*n* = 1)	Others (*n* = 3)
Florencio, et al., (2001)	**✓**			
Fernald & Neufeld (2007)	**✓**			**✓** ^α^
Severi & Moratorio, (2014)		**✓**		
Kinyoki, et al., (2016)		**✓**		
Rachmi, et al., (2016)		**✓**		**✓** ^∞^
Saaka & Galaa, (2016)		**✓**		
Zhang, et al., (2016)			**✓**	
Mgongo, et al., (2017)		**✓**		
Minh Do, et al., (2018)		**✓**		
Zhang, et al., (2018)		**✓**		
Fongar et al., (2019)		**✓**		
Garanne, et al., (2019)	**✓**			
Islam & Biswas, (2019)		**✓**		
Varghese & Stein (2019)		**✓**		
Yasmin, et al., (2019)		**✓**		
Ferreira, (2020)		**✓**		
Benedict, et al., (2020)		**✓**		
Farah, et al. (2021)		**✓**		
Zhang, et al., (2021)		**✓**		**✓**^**⁎**^
Roba, et al., (2021)		**✓**		
Khaliq, et al., (2021)		**✓**		

∞ = Pan and LMS growth program, α = Anthropometric standardization reference manual and Standardization of quantitative epidemiological methods in the field, * = WHO child growth reference-2007

Fourteen studies had adequately defined reporting criteria [29–31, 33, 37, 44–46, 48, 50]. These studies assessed the relationship of predictor and outcome variables using appropriate statistical tests, such as multivariate or multinomial regression that adjust for confounding effects of each variable to avoid erroneous findings and invalid conclusions [55].

A total of nine studies reported response rates. The lowest response rate observed was 23%, where 173 out of 750 households of mother-infant dyad participated, and no attempts were made to reduce the low response rate [35]. In five studies, the response rate was over 90% [33, 38, 47–49, 54], while in the remaining three studies response rates observed were 76% [29], 81% [31], and 82% [30], respectively. Non-response rates were adjusted for sampling weights in one study [29].

## Discussion

Coexisting forms of malnutrition (CFM) are a relatively novel concept of malnutrition, first identified in the 2014 Global Nutrition Report [41]. As a result, most of the studies included in this review were published after 2015. To date, CFM is not well-researched, and the prevalence, trends, and determinants of CFM have only been assessed in a few geographical regions of Asia, Africa, and South America.

CFM represents the simultaneous presence of more than one nutritional disorder in an individual. This review identified more than 10 different types of CFM. There are certain types of CFM experienced by both children as well as by adults, such as undernutrition with MRM and overnutrition with MRM. MRM may exist in any individual irrespective of body weight and BMI [56–58]. In the pediatric population, additional types of CFM exist, such as the coexistence of undernutrition (presence of stunting, and/or wasting and/or underweight and/or all) and coexistence of undernutrition and overnutrition, commonly referred to as the coexistence of stunting with overweight/obesity or Double Burden of Malnutrition (DBM) in an individual.

Like standalone forms of malnutrition, CFM can be assessed using conventional assessment methods, such as anthropometry, biochemical testing, clinical examination, and dietary recall [13–15]. The Global Nutrition Report is the only international report where the prevalence of two basic types of CFM are explicitly presented: coexistence of stunting with wasting and coexistence of stunting with overweight/obesity at global level [41–43]. Similarly, various studies conducted by individual researchers presented the prevalence of various forms of CFM using national and regional health and nutrition datasets. Globally, the Demographic and Health Surveys (DHS), and Multiple Indicator Cluster Surveys (MICS) are two major datasets, which collect health and nutrition related information from women and children at national and regional level respectively. These datasets are useful resources for examining the prevalence, trends, and determinants of various types of CFM in children under 5 years of age. Still, the prevalence, trends, and determinants of CFM and its various types has not been presented in the DHS and MICS reports [59, 60].

This review identified various types of CFM in neonates, infants, and children using a variety of anthropometric indices, such as, HAZ, WHZ, WAZ, BAZ, BMI, MUAC, and skin folds. These nutritional indicators were also proposed by WHO, CDC, and the Food and Nutrition Technical Assistance (FANTA) project for assessing various types of malnutrition in children [61]. Among different anthropometric indices, WAZ, WHZ, and BAZ measured weight for length/height and age. Hence, these anthropometric indices assessed wasting, underweight, and overweight/obesity in children. The WHO, CDC, and FANTA discouraged the use of WAZ for assessing overweight/obesity, as WAZ only represents weight deficit according to the age of the children [36]. Thus, BAZ and WHZ are recommended anthropometric indices for determining the overweight/obesity and acute forms of malnutrition (wasting) [36, 61]. Except Varghese & Stein (2019), most of the studies included in this review either used WHZ or BAZ for assessing overweight/obesity [38]. The BAZ produced higher estimates of overweight/obesity in children below 5 years of age than the WHZ [48] and maybe a less reliable anthropometric indicator than the WHZ for assessing pediatric obesity.

In this review, the nutritional status of all children was assessed using manual anthropometric methods. Technological methods for the assessment of nutrition statuses, such as Air Displacement Plethysmography (ADP), Computed Tomography (CT) scan, Dual X-ray Absorptiometry (DXA) scan, and Magnetic Resonance Imaging (MRI) were not employed in any study. Technological methods are more reliable than the manual anthropometric methods for the assessment of nutritional status but are not suitable for large epidemiological studies due to cost and potential side effects [62]. All the studies included in this review assessed child nutritional status through z-scores. No efforts were made to validate the manual anthropometric accuracy by using other manual anthropometric methods, such as triceps skinfolds thickness, subscapular skinfolds thickness, MUAC, or circumference measurement of head, hips, and waist [13, 62]. Any imprecision in the assessment of nutritional status from the included studies is unknown. Further research is needed that combines different anthropometric methods using standardized protocols for estimating and minimizing potential inaccuracies associated with manual anthropometric methods.

The global prevalence of various forms of CFM is not evenly distributed. This review identified a high prevalence of coexistence of stunting with overweight/obesity in Europe, and high coexistence of wasting with stunting from Asian and African countries [41]. Worldwide, there is no defined threshold for determining a high population prevalence of CFM. However, the Technical Expert Advisory Groups on Malnutrition (TEAM) have defined the prevalence of over 20% stunting, and/or over 10% wasting and/or over 10% overweight/obesity cases to reflect a high prevalence of malnutrition [63]. Different reports confirmed a high prevalence of undernutrition (stunting, wasting, and underweight) among Asian and African children, while children living in Europe, Oceania, and America are generally facing issues related to overnutrition (overweight/obesity) [64–66]. Despite having global statistics regarding the prevalence of stunting, wasting, underweight, and overweight/obesity, few studies reported the prevalence of various types of CFM, and these studies were confined to a few countries of Asia, Africa, and South America. A more exhaustive examination of the regional prevalence of various types of CFM will help to identify the most vulnerable regions for specific types of CFM.

A limited number of studies described the trends of CFM over time. Most reported a decrease in the coexistence of stunting with overweight/obesity and coexistence of wasting with stunting, possibly due to the decreased prevalence of undernutrition, such as stunting, and wasting [67]. In contrast, a study conducted in Indonesia showed increasing coexistence of stunting with overweight/obesity [33] which might be attributed to nutritional transition. The nutritional transition is characterized by a decline in the prevalence of undernutrition (stunting, wasting, and underweight), with the simultaneous proliferation of overnutrition (overweight/obesity) over time [67]. Changes in dietary practices from low fat, and high fibre diet to high energy and low fibre diet serve as an important determinant for nutritional transition [68]. The issues related to nutritional transition have penetrated the geographical boundaries of every country, resulting in the coexistence of stunting with overweight/obesity, commonly referred to as DBM at an individual level. The DBM is a global issue and requires double action, such as simultaneous management of undernutrition and overnutrition for the prevention and control of malnutrition [67–70]. Popkin et al. published a series that described the dynamics, determinants and economic effects of DBM [20, 71, 72]. Still, the evidence regarding the trends of various types of CFM is underdeveloped, and more research is needed to investigate trends of specific types of CFM at the global, national, and regional levels.

Most of the studies included in this review assessed the determinants of coexistence of stunting with overweight/obesity, while the determinants for other types of CFM remain largely unknown. Findings from this review suggest that young maternal age and short maternal height may increase the risk of coexistence of stunting with overweight/obesity [73, 74]. Each centimetre decrease in maternal height is associated with a decrease in offspring height by 0.25 to 0.48 cm [74]. Besides stunting, short maternal height produces a drastic effect on birth weight, birth size, muscle wasting, and death [74, 75]. The risk of stunting among the offspring of adolescent mothers was eight times higher than the offspring of adult mothers [76] and stunting itself increased the odds of overweight/obesity by 7.8 (95% CI: 5.7 to 10.7) [77, 78]. Children born to adolescent mothers are often low birth weight (LBW) and have stunted growth [79, 80]. In adolescent pregnancy, the mother and the growing foetus compete for nutrients, and this nutritional competition results in adverse health consequences, such as LBW, premature birth, anaemia, eclampsia, sepsis, and other pregnancy-related complications [81, 82]. Thus, global legislation and its proper execution against the practices of early marriages, and adolescent pregnancies can curtail the intergenerational consequences of CFM [83].

Certain factors were found to produce a protective effect on the coexistence of stunting with overweight/obesity. It is evident from this review that urbanization reduces the risk of all types of undernutrition (stunting, wasting, underweight) and the coexistence of stunting with overweight/obesity. It has been previously established that urbanization is associated with overnutrition (overweight/obesity) while living in pastoral areas is linked with undernutrition (stunting, wasting, underweight) [84, 85]. Most of the studies reviewed to support a risk-reducing effect of urbanization on the coexistence of stunting with overweight/obesity, although the mechanisms for an effect of rapid urbanization on COS are unclear. According to Yasmin et al., (2019), stunting in children is an important determinant for the coexistence of stunting with overweight/obesity [31]. Further research is needed to understand the relationship of various types of CFM with different types of standalone forms of malnutrition.

Evidence for the role of different food groups in CFM risk and protection is limited. This review identified two studies that assessed the relationship of various types of food and micronutrient supplementation with CFM. In general, no association of CFM with the intake of specific foods was reported. There is a global influx of overweight/obesity together with undernutrition and micronutrient deficiency at the individual, household, and community level, due to changes in the dietary habits from low fat and high fiber diet too high caloric, non-nutritious, low fiber and high-fat food [86, 87]. Studies conducted previously have shown that protein consumption can be effectively increased for the management of moderately acute malnourished (MAM) and severely acute malnourished (SAM) children [88]. Similarly, fruits and vegetables are rich sources of fibres, vitamins, and minerals that protect an individual from micronutrient deficiencies [89, 90]. Micronutrients are essential for growth, nourishment, and appropriate body functions, and their deficiency results in a wide range of neurocognitive and physical disorders, such as rickets in children and osteomalacia in adults [91–93]. For reducing CFM, it is essential to know about the caloric requirement and dietary benefits of all types of food to alleviate MRM together with undernutrition and/or overnutrition.

### Strengths and limitations

To the best of our knowledge, this is the first review, which has explicitly presented the evidence regarding the prevalence, determinants, and trends of CFM at an individual level. The current review used a range of databases for a systematic review of the literature, consultation with an expert librarian for the Peer Review of Electronic Search Strategy (PRESS), and consultation with stakeholders for unpublished literature access. Most of the studies included in this review used WHO child growth standards, which has a reliability coefficient over 95% for most of the anthropometric indices [94].

A limitation of this study is that only certain types of CFM were measured, because of the exclusion of studies that solely discussed the coexistence of micronutrient-related malnutrition (CMRM). Included studies examined MRM (anemia) with under/overnutrition, while other types of MRM were not discussed, such as deficiency/intoxication of vitamin-A, vitamin-D, folic acid, iodine, and calcium.

In this review, studies conducted in an institutional setting were excluded to narrow the scope of the review. Therefore, the burden of CFM among individuals with other illnesses that resulted in hospitalisation has not been captured. This could mean that the true prevalence of CFM may be much higher than what has been reported in this review.

## Conclusion

Several types of CFM may exist at the individual level, but evidence regarding the prevalence, determinants, and trends for each type of CFM is scarce. Apart from the coexistence of stunting with overweight/obesity, the determinants of other types of CFM are unclear. Like standalone forms of malnutrition, CFM assessment should be incorporated into standardised reporting from national nutrition surveillance and demographic health survey programs. Efforts towards assessing the prevalence, trends, and determinants of various types of CFM in both children and adults, using similar assessment methods to that of standalone forms of malnutrition, could rapidly advance the capacity of global, regional, and local policy and health service responses for addressing the burden of CFM through targeted and effective strategies for their prevention and control.

## Supplementary Information

**Additional file 1.**

**Additional file 2.**

**Additional file 3.**

**Additional file 4.**

## Data Availability

Not applicable.
