# Supplementary material for: A review of the prevalence, trends, and determinants of coexisting forms of malnutrition in neonates, infants, and children
Source: BMC Public Health. 2022 May 3;22:879. doi: 10.1186/s12889-022-13098-9 (PMC9063291; doi:10.1186/s12889-022-13098-9)
Supplement: Supplementary file 2 — Additional file 2. [file 12889_2022_13098_MOESM2_ESM.docx]

**Supplementary file 2**

**JBI CRITICAL APPRAISAL CHECKLIST FOR PREVALENCE STUDIES**

**Author________________________________ Year_________ Record Number_______**

**Assessment and categorization of different factors from JBI scale of prevalence studies**

| **Item No.** | **Explanation** | **Categorization** |
| --- | --- | --- |
| 1 | Was the sample frame appropriate to address the target population? | Selection |
| 2 | Were study participants sampled in an appropriate way? | Selection |
| 3 | Was the sample size adequate? | Selection |
| 4 | Were the study subjects and the setting described in detail? | Selection |
| 5 | Was the data analysis conducted with sufficient coverage of the identified sample? | Selection |
| 6 | Were valid methods used for the identification of the condition? | Measurement |
| 7 | Was the condition measured in a standard, reliable way for all participants? | Measurement |
| 8 | Was there appropriate statistical analysis? | Reporting |
| 9 | Was the response rate adequate, and if not, was the low response rate managed appropriately? | Attrition |

**Rating:**

The following table is showing minimum number of stars required in each factor

| Studies rating | Selection | Measurement | Reporting | Attrition |
| --- | --- | --- | --- | --- |
| Good | 4 to 5 | 1.5 to 2 | 1 | 1 |
| Fair | 2 to 3.5 | 0.5 to 1 | 0.5 | 0.5 |
| Poor | 0 to 1.5 | 0 | 0 | 0 |

**Star obtained:**

| Selection: |  |  |
| --- | --- | --- |
| Measurement: |  |  |
| Reporting: |  |  |
| Attrition: |  |  |
| **Overall:** |  |  |

**JBI CRITICAL APPRAISAL CHECKLIST FOR PREVALENCE STUDIES**

**Author ____________**Florencio, et al., **____________ Year_**2001

| **Item No.** | **Explanation** | **Categorization** | **Stars obtained** |
| --- | --- | --- | --- |
| 1 | Was the sample frame appropriate to address the target population? | Selection | 1 |
| 2 | Were study participants sampled in an appropriate way? | Selection | 0 |
| 3 | Was the sample size adequate? | Selection | 0 |
| 4 | Were the study subjects and the setting described in detail? | Selection | 0.5 |
| 5 | Was the data analysis conducted with sufficient coverage of the identified sample? | Selection | 0 |
| 6 | Were valid methods used for the identification of the condition? | Measurement | 1 |
| 7 | Was the condition measured in a standard, reliable way for all participants? | Measurement | 1 |
| 8 | Was there appropriate statistical analysis? | Reporting | 0.5 |
| 9 | Was the response rate adequate, and if not, was the low response rate managed appropriately? | Attrition | 0 |

**Rating:**

The following table is showing minimum number of stars required in each factor

| Studies rating | Selection | Measurement | Reporting | Attrition |
| --- | --- | --- | --- | --- |
| Good | 4 to 5 | 1.5 to 2 | 1 | 1 |
| Fair | 2 to 3.5 | 0.5 to 1 | 0.5 | 0.5 |
| Poor | 0 to 1.5 | 0 | 0 | 0 |

**Star obtained:**

| Selection: | 1.5 | Poor |
| --- | --- | --- |
| Measurement: | 2 | Good |
| Reporting: | 0.5 | Fair |
| Attrition: | 0 | Poor |
| **Overall:** | **4** | **Poor** |

**JBI CRITICAL APPRAISAL CHECKLIST FOR PREVALENCE STUDIES**

**Author ____________**Fernald & Neufeld **____________ Year_**2007

| **Item No.** | **Explanation** | **Categorization** | **Stars obtained** |
| --- | --- | --- | --- |
| 1 | Was the sample frame appropriate to address the target population? | Selection | 1 |
| 2 | Were study participants sampled in an appropriate way? | Selection | 0.5 |
| 3 | Was the sample size adequate? | Selection | 1 |
| 4 | Were the study subjects and the setting described in detail? | Selection | 1 |
| 5 | Was the data analysis conducted with sufficient coverage of the identified sample? | Selection | 0.5 |
| 6 | Were valid methods used for the identification of the condition? | Measurement | 1 |
| 7 | Was the condition measured in a standard, reliable way for all participants? | Measurement | 1 |
| 8 | Was there appropriate statistical analysis? | Reporting | 1 |
| 9 | Was the response rate adequate, and if not, was the low response rate managed appropriately? | Attrition | 1 |

**Rating:**

The following table is showing minimum number of stars required in each factor

| Studies rating | Selection | Measurement | Reporting | Attrition |
| --- | --- | --- | --- | --- |
| Good | 4 to 5 | 1.5 to 2 | 1 | 1 |
| Fair | 2 to 3.5 | 0.5 to 1 | 0.5 | 0.5 |
| Poor | 0 to 1.5 | 0 | 0 | 0 |

**Star obtained:**

| Selection: | 4 | Good |
| --- | --- | --- |
| Measurement: | 2 | Good |
| Reporting: | 1 | Good |
| Attrition: | 1 | Good |
| **Overall:** | **8** | **Good** |

**JBI CRITICAL APPRAISAL CHECKLIST FOR PREVALENCE STUDIES**

**Author ____________**Severi & Moratorio **____________ Year_**2014

| **Item No.** | **Explanation** | **Categorization** | **Stars obtained** |
| --- | --- | --- | --- |
| 1 | Was the sample frame appropriate to address the target population? | Selection | 0.5 |
| 2 | Were study participants sampled in an appropriate way? | Selection | 0.5 |
| 3 | Was the sample size adequate? | Selection | 1 |
| 4 | Were the study subjects and the setting described in detail? | Selection | 0.5 |
| 5 | Was the data analysis conducted with sufficient coverage of the identified sample? | Selection | 0.5 |
| 6 | Were valid methods used for the identification of the condition? | Measurement | 0 |
| 7 | Was the condition measured in a standard, reliable way for all participants? | Measurement | 0.5 |
| 8 | Was there appropriate statistical analysis? | Reporting | 0 |
| 9 | Was the response rate adequate, and if not, was the low response rate managed appropriately? | Attrition | 0.5 |

**Rating:**

The following table is showing minimum number of stars required in each factor

| Studies rating | Selection | Measurement | Reporting | Attrition |
| --- | --- | --- | --- | --- |
| Good | 4 to 5 | 1.5 to 2 | 1 | 1 |
| Fair | 2 to 3.5 | 0.5 to 1 | 0.5 | 0.5 |
| Poor | 0 to 1.5 | 0 | 0 | 0 |

**Star obtained:**

| Selection: | 3 | Fair |
| --- | --- | --- |
| Measurement: | 0.5 | Fair |
| Reporting: | 0 | Poor |
| Attrition: | 0.5 | Fair |
| **Overall:** | **4** | **Poor** |

**JBI CRITICAL APPRAISAL CHECKLIST FOR PREVALENCE STUDIES**

**Author ____________**Kinyoki, el al., **____________ Year_**2016

| **Item No.** | **Explanation** | **Categorization** | **Stars obtained** |
| --- | --- | --- | --- |
| 1 | Was the sample frame appropriate to address the target population? | Selection | 1 |
| 2 | Were study participants sampled in an appropriate way? | Selection | 0.5 |
| 3 | Was the sample size adequate? | Selection | 1 |
| 4 | Were the study subjects and the setting described in detail? | Selection | 0.5 |
| 5 | Was the data analysis conducted with sufficient coverage of the identified sample? | Selection | 0.5 |
| 6 | Were valid methods used for the identification of the condition? | Measurement | 0 |
| 7 | Was the condition measured in a standard, reliable way for all participants? | Measurement | 1 |
| 8 | Was there appropriate statistical analysis? | Reporting | 0.5 |
| 9 | Was the response rate adequate, and if not, was the low response rate managed appropriately? | Attrition | 0 |

**Rating:**

The following table is showing minimum number of stars required in each factor

| Studies rating | Selection | Measurement | Reporting | Attrition |
| --- | --- | --- | --- | --- |
| Good | 4 to 5 | 1.5 to 2 | 1 | 1 |
| Fair | 2 to 3.5 | 0.5 to 1 | 0.5 | 0.5 |
| Poor | 0 to 1.5 | 0 | 0 | 0 |

**Star obtained:**

| Selection: | 3.5 | Fair |
| --- | --- | --- |
| Measurement: | 1 | Fair |
| Reporting: | 1 | Good |
| Attrition: | 0 | Poor |
| **Overall:** | **5.5** | **Fair** |

**JBI CRITICAL APPRAISAL CHECKLIST FOR PREVALENCE STUDIES**

**Author ____________**Rachmi, el al., **____________ Year_**2016

| **Item No.** | **Explanation** | **Categorization** | **Stars obtained** |
| --- | --- | --- | --- |
| 1 | Was the sample frame appropriate to address the target population? | Selection | 0.5 |
| 2 | Were study participants sampled in an appropriate way? | Selection | 1 |
| 3 | Was the sample size adequate? | Selection | 1 |
| 4 | Were the study subjects and the setting described in detail? | Selection | 1 |
| 5 | Was the data analysis conducted with sufficient coverage of the identified sample? | Selection | 0.5 |
| 6 | Were valid methods used for the identification of the condition? | Measurement | 0 |
| 7 | Was the condition measured in a standard, reliable way for all participants? | Measurement | 1 |
| 8 | Was there appropriate statistical analysis? | Reporting | 0.5 |
| 9 | Was the response rate adequate, and if not, was the low response rate managed appropriately? | Attrition | 1 |

**Rating:**

The following table is showing minimum number of stars required in each factor

| Studies rating | Selection | Measurement | Reporting | Attrition |
| --- | --- | --- | --- | --- |
| Good | 4 to 5 | 1.5 to 2 | 1 | 1 |
| Fair | 2 to 3.5 | 0.5 to 1 | 0.5 | 0.5 |
| Poor | 0 to 1.5 | 0 | 0 | 0 |

**Star obtained:**

| Selection: | 4 | Good |
| --- | --- | --- |
| Measurement: | 1 | Fair |
| Reporting: | 1 | Good |
| Attrition: | 1 | Good |
| **Overall:** | **7** | **Good** |

***JBI CRITICAL APPRAISAL CHECKLIST FOR PREVALENCE STUDIES***

***Author ______****Saaka & Gala.,* ***_______ Year__****2016____*

***Assessment and categorization of different factors from JBI scale of prevalence studies***

| ***Item No.*** | ***Explanation*** | ***Categorization*** |
| --- | --- | --- |
| *1* | *Was the sample frame appropriate to address the target population?* | *1* |
| *2* | *Were study participants sampled in an appropriate way?* | *1* |
| *3* | *Was the sample size adequate?* | *1* |
| *4* | *Were the study subjects and the setting described in detail?* | *1* |
| *5* | *Was the data analysis conducted with sufficient coverage of the identified sample?* | *0.5* |
| *6* | *Were valid methods used for the identification of the condition?* | *1* |
| *7* | *Was the condition measured in a standard, reliable way for all participants?* | *0.5* |
| *8* | *Was there appropriate statistical analysis?* | *1* |
| *9* | *Was the response rate adequate, and if not, was the low response rate managed appropriately?* | *1* |

**Rating:**

The following table is showing minimum number of stars required in each factor

| Studies rating | Selection | Measurement | Reporting | Attrition |
| --- | --- | --- | --- | --- |
| Good | 4 to 5 | 1.5 to 2 | 1 | 1 |
| Fair | 2 to 3.5 | 0.5 to 1 | 0.5 | 0.5 |
| Poor | 0 to 1.5 | 0 | 0 | 0 |

***Star obtained:***

| *Selection:* | *4.5* |
| --- | --- |
| *Measurement:* | *1.5* |
| *Reporting:* | *1* |
| *Attrition:* | *1* |
| ***Overall:*** | ***8*** |

**JBI CRITICAL APPRAISAL CHECKLIST FOR PREVALENCE STUDIES**

**Author ____________**Zhang, et al., **____________ Year_**2016

| **Item No.** | **Explanation** | **Categorization** | **Stars obtained** |
| --- | --- | --- | --- |
| 1 | Was the sample frame appropriate to address the target population? | Selection | 1 |
| 2 | Were study participants sampled in an appropriate way? | Selection | 1 |
| 3 | Was the sample size adequate? | Selection | 1 |
| 4 | Were the study subjects and the setting described in detail? | Selection | 1 |
| 5 | Was the data analysis conducted with sufficient coverage of the identified sample? | Selection | 0.5 |
| 6 | Were valid methods used for the identification of the condition? | Measurement | 1 |
| 7 | Was the condition measured in a standard, reliable way for all participants? | Measurement | 1 |
| 8 | Was there appropriate statistical analysis? | Reporting | 1 |
| 9 | Was the response rate adequate, and if not, was the low response rate managed appropriately? | Attrition | 0 |

**Rating:**

The following table is showing minimum number of stars required in each factor

| Studies rating | Selection | Measurement | Reporting | Attrition |
| --- | --- | --- | --- | --- |
| Good | 4 to 5 | 1.5 to 2 | 1 | 1 |
| Fair | 2 to 3.5 | 0.5 to 1 | 0.5 | 0.5 |
| Poor | 0 to 1.5 | 0 | 0 | 0 |

**Star obtained:**

| Selection: | 4.5 | Good |
| --- | --- | --- |
| Measurement: | 2 | Good |
| Reporting: | 1 | Good |
| Attrition: | 0 | Poor |
| **Overall:** | **7.5** | **Good** |

**JBI CRITICAL APPRAISAL CHECKLIST FOR PREVALENCE STUDIES**

**Author _________**Mgongo, et al.,_________ **Year___**2017**______**

**Assessment and categorization of different factors from JBI scale of prevalence studies**

| **Item No.** | **Explanation** | **Categorization** |
| --- | --- | --- |
| 1 | Was the sample frame appropriate to address the target population? | 1 |
| 2 | Were study participants sampled in an appropriate way? | 1 |
| 3 | Was the sample size adequate? | 1 |
| 4 | Were the study subjects and the setting described in detail? | 1 |
| 5 | Was the data analysis conducted with sufficient coverage of the identified sample? | 1 |
| 6 | Were valid methods used for the identification of the condition? | 1 |
| 7 | Was the condition measured in a standard, reliable way for all participants? | 1 |
| 8 | Was there appropriate statistical analysis? | 1 |
| 9 | Was the response rate adequate, and if not, was the low response rate managed appropriately? | 0.5 |

**Rating:**

The following table is showing minimum number of stars required in each factor

| Studies rating | Selection | Measurement | Reporting | Attrition |
| --- | --- | --- | --- | --- |
| Good | 4 to 5 | 1.5 to 2 | 1 | 1 |
| Fair | 2 to 3.5 | 0.5 to 1 | 0.5 | 0.5 |
| Poor | 0 to 1.5 | 0 | 0 | 0 |

**Star obtained:**

| Selection: | 5 | Good |
| --- | --- | --- |
| Measurement: | 2 | Good |
| Reporting: | 1 | Good |
| Attrition: | 0.5 | Fair |
| **Overall:** | **8.5** | **Good** |

**JBI CRITICAL APPRAISAL CHECKLIST FOR PREVALENCE STUDIES**

**Author ____________**Zhang, et al., **____________ Year_**2018

| **Item No.** | **Explanation** | **Categorization** | **Stars obtained** |
| --- | --- | --- | --- |
| 1 | Was the sample frame appropriate to address the target population? | Selection | 1 |
| 2 | Were study participants sampled in an appropriate way? | Selection | 1 |
| 3 | Was the sample size adequate? | Selection | 1 |
| 4 | Were the study subjects and the setting described in detail? | Selection | 1 |
| 5 | Was the data analysis conducted with sufficient coverage of the identified sample? | Selection | 0 |
| 6 | Were valid methods used for the identification of the condition? | Measurement | 1 |
| 7 | Was the condition measured in a standard, reliable way for all participants? | Measurement | 1 |
| 8 | Was there appropriate statistical analysis? | Reporting | 1 |
| 9 | Was the response rate adequate, and if not, was the low response rate managed appropriately? | Attrition | 1 |

**Rating:**

The following table is showing minimum number of stars required in each factor

| Studies rating | Selection | Measurement | Reporting | Attrition |
| --- | --- | --- | --- | --- |
| Good | 4 to 5 | 1.5 to 2 | 1 | 1 |
| Fair | 2 to 3.5 | 0.5 to 1 | 0.5 | 0.5 |
| Poor | 0 to 1.5 | 0 | 0 | 0 |

**Star obtained:**

| Selection: | 4 | Good |
| --- | --- | --- |
| Measurement: | 2 | Good |
| Reporting: | 1 | Good |
| Attrition: | 1 | Good |
| **Overall:** | **8** | **Good** |

**JBI CRITICAL APPRAISAL CHECKLIST FOR PREVALENCE STUDIES**

**Author ____________**Fongar, el al., **____________ Year_**2019

| **Item No.** | **Explanation** | **Categorization** | **Stars obtained** |
| --- | --- | --- | --- |
| 1 | Was the sample frame appropriate to address the target population? | Selection | 1 |
| 2 | Were study participants sampled in an appropriate way? | Selection | 1 |
| 3 | Was the sample size adequate? | Selection | 1 |
| 4 | Were the study subjects and the setting described in detail? | Selection | 1 |
| 5 | Was the data analysis conducted with sufficient coverage of the identified sample? | Selection | 0 |
| 6 | Were valid methods used for the identification of the condition? | Measurement | 1 |
| 7 | Was the condition measured in a standard, reliable way for all participants? | Measurement | 1 |
| 8 | Was there appropriate statistical analysis? | Reporting | 0.5 |
| 9 | Was the response rate adequate, and if not, was the low response rate managed appropriately? | Attrition | 0 |

**Rating:**

The following table is showing minimum number of stars required in each factor

| Studies rating | Selection | Measurement | Reporting | Attrition |
| --- | --- | --- | --- | --- |
| Good | 4 to 5 | 1.5 to 2 | 1 | 1 |
| Fair | 2 to 3.5 | 0.5 to 1 | 0.5 | 0.5 |
| Poor | 0 to 1.5 | 0 | 0 | 0 |

**Star obtained:**

| Selection: | 4 | Good |
| --- | --- | --- |
| Measurement: | 2 | Good |
| Reporting: | 0.5 | Fair |
| Attrition: | 0 | Poor |
| **Overall:** | **6.5** | **Fair** |

**JBI CRITICAL APPRAISAL CHECKLIST FOR PREVALENCE STUDIES**

**Author _______**Islam & Biswas**_____________ Year_____**2019**____**

**Assessment and categorization of different factors from JBI scale of prevalence studies**

| **Item No.** | **Explanation** | **Categorization** |
| --- | --- | --- |
| 1 | Was the sample frame appropriate to address the target population? | 1 |
| 2 | Were study participants sampled in an appropriate way? | 1 |
| 3 | Was the sample size adequate? | 1 |
| 4 | Were the study subjects and the setting described in detail? | 1 |
| 5 | Was the data analysis conducted with sufficient coverage of the identified sample? | 1 |
| 6 | Were valid methods used for the identification of the condition? | 1 |
| 7 | Was the condition measured in a standard, reliable way for all participants? | 1 |
| 8 | Was there appropriate statistical analysis? | 1 |
| 9 | Was the response rate adequate, and if not, was the low response rate managed appropriately? | 0.5 |

**Rating:**

The following table is showing minimum number of stars required in each factor

| Studies rating | Selection | Measurement | Reporting | Attrition |
| --- | --- | --- | --- | --- |
| Good | 4 to 5 | 1.5 to 2 | 1 | 1 |
| Fair | 2 to 3.5 | 0.5 to 1 | 0.5 | 0.5 |
| Poor | 0 to 1.5 | 0 | 0 | 0 |

**Star obtained:**

| Selection: | 5 | Good |
| --- | --- | --- |
| Measurement: | 2 | Good |
| Reporting: | 1 | Good |
| Attrition: | 0.5 | Fair |
| **Overall:** | **8.5** | **Good** |

**JBI CRITICAL APPRAISAL CHECKLIST FOR PREVALENCE STUDIES**

**Author ____________**Varghese & Stein **____________ Year_**2019

| **Item No.** | **Explanation** | **Categorization** | **Stars obtained** |
| --- | --- | --- | --- |
| 1 | Was the sample frame appropriate to address the target population? | Selection | 1 |
| 2 | Were study participants sampled in an appropriate way? | Selection | 1 |
| 3 | Was the sample size adequate? | Selection | 1 |
| 4 | Were the study subjects and the setting described in detail? | Selection | 1 |
| 5 | Was the data analysis conducted with sufficient coverage of the identified sample? | Selection | 0.5 |
| 6 | Were valid methods used for the identification of the condition? | Measurement | 0.5 |
| 7 | Was the condition measured in a standard, reliable way for all participants? | Measurement | 1 |
| 8 | Was there appropriate statistical analysis? | Reporting | 0 |
| 9 | Was the response rate adequate, and if not, was the low response rate managed appropriately? | Attrition | 1 |

**Rating:**

The following table is showing minimum number of stars required in each factor

| Studies rating | Selection | Measurement | Reporting | Attrition |
| --- | --- | --- | --- | --- |
| Good | 4 to 5 | 1.5 to 2 | 1 | 1 |
| Fair | 2 to 3.5 | 0.5 to 1 | 0.5 | 0.5 |
| Poor | 0 to 1.5 | 0 | 0 | 0 |

**Star obtained:**

| Selection: | 4.5 | Good |
| --- | --- | --- |
| Measurement: | 1.5 | Fair |
| Reporting: | 0 | Poor |
| Attrition: | 1 | Good |
| **Overall:** | **7** | **Good** |

***JBI CRITICAL APPRAISAL CHECKLIST FOR PREVALENCE STUDIES***

***Author _______****Yasmin, et al.,* ***_______ Year__****2019____*

***Assessment and categorization of different factors from JBI scale of prevalence studies***

| ***Item No.*** | ***Explanation*** | ***Categorization*** |
| --- | --- | --- |
| *1* | *Was the sample frame appropriate to address the target population?* | *1* |
| *2* | *Were study participants sampled in an appropriate way?* | *1* |
| *3* | *Was the sample size adequate?* | *1* |
| *4* | *Were the study subjects and the setting described in detail?* | *1* |
| *5* | *Was the data analysis conducted with sufficient coverage of the identified sample?* | *0.5* |
| *6* | *Were valid methods used for the identification of the condition?* | *1* |
| *7* | *Was the condition measured in a standard, reliable way for all participants?* | *0.5* |
| *8* | *Was there appropriate statistical analysis?* | *1* |
| *9* | *Was the response rate adequate, and if not, was the low response rate managed appropriately?* | *1* |

**Rating:**

The following table is showing minimum number of stars required in each factor

| Studies rating | Selection | Measurement | Reporting | Attrition |
| --- | --- | --- | --- | --- |
| Good | 4 to 5 | 1.5 to 2 | 1 | 1 |
| Fair | 2 to 3.5 | 0.5 to 1 | 0.5 | 0.5 |
| Poor | 0 to 1.5 | 0 | 0 | 0 |

***Star obtained:***

| *Selection:* | *4.5* |
| --- | --- |
| *Measurement:* | *1.5* |
| *Reporting:* | *1* |
| *Attrition:* | *1* |
| ***Overall:*** | ***8*** |

***JBI CRITICAL APPRAISAL CHECKLIST FOR PREVALENCE* STUDIES**

**Author _____**Ferreira**_________ Year___**2020**____**

**Assessment and categorization of different factors from JBI scale of prevalence studies**

| **Item No.** | **Explanation** | **Categorization** |
| --- | --- | --- |
| 1 | Was the sample frame appropriate to address the target population? | 1 |
| 2 | Were study participants sampled in an appropriate way? | 1 |
| 3 | Was the sample size adequate? | 1 |
| 4 | Were the study subjects and the setting described in detail? | 1 |
| 5 | Was the data analysis conducted with sufficient coverage of the identified sample? | 1 |
| 6 | Were valid methods used for the identification of the condition? | 1 |
| 7 | Was the condition measured in a standard, reliable way for all participants? | 1 |
| 8 | Was there appropriate statistical analysis? | 0.5 |
| 9 | Was the response rate adequate, and if not, was the low response rate managed appropriately? | 1 |

**Rating:**

The following table is showing minimum number of stars required in each factor

| Studies rating | Selection | Measurement | Reporting | Attrition |
| --- | --- | --- | --- | --- |
| Good | 4 to 5 | 1.5 to 2 | 1 | 1 |
| Fair | 2 to 3.5 | 0.5 to 1 | 0.5 | 0.5 |
| Poor | 0 to 1.5 | 0 | 0 | 0 |

**Star obtained:**

| Selection: | 5 | Good |
| --- | --- | --- |
| Measurement: | 2 | Good |
| Reporting: | 0.5 | Fair |
| Attrition: | 0 | Poor |
| **Overall:** | **7.5** | **Good** |

**JBI CRITICAL APPRAISAL CHECKLIST FOR PREVALENCE STUDIES**

**Author ________**Benedict, et al.,**__________ Year_ 2020____**

| **Item No.** | **Explanation** | **Categorization** | **Stars obtained** |
| --- | --- | --- | --- |
| 1 | Was the sample frame appropriate to address the target population? | Selection | 1 |
| 2 | Were study participants sampled in an appropriate way? | Selection | 1 |
| 3 | Was the sample size adequate? | Selection | 1 |
| 4 | Were the study subjects and the setting described in detail? | Selection | 1 |
| 5 | Was the data analysis conducted with sufficient coverage of the identified sample? | Selection | 1 |
| 6 | Were valid methods used for the identification of the condition? | Measurement | 1 |
| 7 | Was the condition measured in a standard, reliable way for all participants? | Measurement | 1 |
| 8 | Was there appropriate statistical analysis? | Reporting | 1 |
| 9 | Was the response rate adequate, and if not, was the low response rate managed appropriately? | Attrition | 1 |

**Rating:**

The following table is showing minimum number of stars required in each factor

| Studies rating | Selection | Measurement | Reporting | Attrition |
| --- | --- | --- | --- | --- |
| Good | 4 to 5 | 1.5 to 2 | 1 | 1 |
| Fair | 2 to 3.5 | 0.5 to 1 | 0.5 | 0.5 |
| Poor | 0 to 1.5 | 0 | 0 | 0 |

**Star obtained:**

| Selection: | 5 | Good |
| --- | --- | --- |
| Measurement: | 2 | Good |
| Reporting: | 1 | Good |
| Attrition: | 1 | Good |
| **Overall:** | **9** | **Good** |

**JBI CRITICAL APPRAISAL CHECKLIST FOR PREVALENCE STUDIES**

**Author_____**Alinoor Mohammd Farah**____ Year___**2021**___**

**Assessment and categorization of different factors from JBI scale of prevalence studies**

| **Item No.** | **Explanation** | **Categorization** |
| --- | --- | --- |
| 1 | Was the sample frame appropriate to address the target population? | 1 |
| 2 | Were study participants sampled in an appropriate way? | 1 |
| 3 | Was the sample size adequate? | 1 |
| 4 | Were the study subjects and the setting described in detail? | 1 |
| 5 | Was the data analysis conducted with sufficient coverage of the identified sample? | 1 |
| 6 | Were valid methods used for the identification of the condition? | 1 |
| 7 | Was the condition measured in a standard, reliable way for all participants? | 1 |
| 8 | Was there appropriate statistical analysis? | 1 |
| 9 | Was the response rate adequate, and if not, was the low response rate managed appropriately? | 1 |

**Rating:**

The following table is showing minimum number of stars required in each factor

| Studies rating | Selection | Measurement | Reporting | Attrition |
| --- | --- | --- | --- | --- |
| Good | 4 to 5 | 1.5 to 2 | 1 | 1 |
| Fair | 2 to 3.5 | 0.5 to 1 | 0.5 | 0.5 |
| Poor | 0 to 1.5 | 0 | 0 | 0 |

**Star obtained:**

| Selection: | 5 |
| --- | --- |
| Measurement: | 2 |
| Reporting: | 1 |
| Attrition: | 1 |
| **Overall:** | **9** |

**JBI CRITICAL APPRAISAL CHECKLIST FOR PREVALENCE STUDIES**

**Author _______Roba, et al.,____________ Year 2021**

| **Item No.** | **Explanation** | **Categorization** | **Stars obtained** |
| --- | --- | --- | --- |
| 1 | Was the sample frame appropriate to address the target population? | Selection | 1 |
| 2 | Were study participants sampled in an appropriate way? | Selection | 1 |
| 3 | Was the sample size adequate? | Selection | 1 |
| 4 | Were the study subjects and the setting described in detail? | Selection | 1 |
| 5 | Was the data analysis conducted with sufficient coverage of the identified sample? | Selection | 1 |
| 6 | Were valid methods used for the identification of the condition? | Measurement | 1 |
| 7 | Was the condition measured in a standard, reliable way for all participants? | Measurement | 1 |
| 8 | Was there appropriate statistical analysis? | Reporting | 1 |
| 9 | Was the response rate adequate, and if not, was the low response rate managed appropriately? | Attrition | 0 |

**Rating:**

The following table is showing minimum number of stars required in each factor

| Studies rating | Selection | Measurement | Reporting | Attrition |
| --- | --- | --- | --- | --- |
| Good | 4 to 5 | 1.5 to 2 | 1 | 1 |
| Fair | 2 to 3.5 | 0.5 to 1 | 0.5 | 0.5 |
| Poor | 0 to 1.5 | 0 | 0 | 0 |

**Star obtained:**

| Selection: | 5 | Good |
| --- | --- | --- |
| Measurement: | 2 | Good |
| Reporting: | 1 | Good |
| Attrition: | 0 | Good |
| **Overall:** | **8** | **Good** |

**JBI CRITICAL APPRAISAL CHECKLIST FOR PREVALENCE STUDIES**

**Author _______Khaliq, et al.,____________ Year 2021**

| **Item No.** | **Explanation** | **Categorization** | **Stars obtained** |
| --- | --- | --- | --- |
| 1 | Was the sample frame appropriate to address the target population? | Selection | 1 |
| 2 | Were study participants sampled in an appropriate way? | Selection | 1 |
| 3 | Was the sample size adequate? | Selection | 1 |
| 4 | Were the study subjects and the setting described in detail? | Selection | 1 |
| 5 | Was the data analysis conducted with sufficient coverage of the identified sample? | Selection | 1 |
| 6 | Were valid methods used for the identification of the condition? | Measurement | 1 |
| 7 | Was the condition measured in a standard, reliable way for all participants? | Measurement | 1 |
| 8 | Was there appropriate statistical analysis? | Reporting | 1 |
| 9 | Was the response rate adequate, and if not, was the low response rate managed appropriately? | Attrition | 0 |

**Rating:**

The following table is showing minimum number of stars required in each factor

| Studies rating | Selection | Measurement | Reporting | Attrition |
| --- | --- | --- | --- | --- |
| Good | 4 to 5 | 1.5 to 2 | 1 | 1 |
| Fair | 2 to 3.5 | 0.5 to 1 | 0.5 | 0.5 |
| Poor | 0 to 1.5 | 0 | 0 | 0 |

**Star obtained:**

| Selection: | 5 | Good |
| --- | --- | --- |
| Measurement: | 2 | Good |
| Reporting: | 1 | Good |
| Attrition: | 0 | Good |
| **Overall:** | **8** | **Good** |
