# Supplementary material for: A review of the prevalence, trends, and determinants of coexisting forms of malnutrition in neonates, infants, and children
Source: BMC Public Health. 2022 May 3;22:879. doi: 10.1186/s12889-022-13098-9 (PMC9063291; doi:10.1186/s12889-022-13098-9)
Supplement: Supplementary file 3 — Additional file 3. [file 12889_2022_13098_MOESM3_ESM.docx]

**Supplementary file 3**

**JBI CRITICAL APPRAISAL CHECKLIST FOR COHORT STUDIES**

**Author________________________________ Year_________**

**Assessment and categorization of different factors from JBI scale of longitudinal studies**

| **Item No.** | **Explanation** | **Categorization** |
| --- | --- | --- |
| 1 | Were the two groups similar and recruited from the same population? | Selection |
| 2 | Were the exposures measured similarly to assign people to both exposed and unexposed groups? | Selection |
| 3 | Was the exposure measured in a valid and reliable way? | Measurement |
| 4 | Were confounding factors identified? | Reporting |
| 5 | Were strategies to deal with confounding factors stated? | Reporting |
| 6 | Were the groups/participants free of the outcome at the start of the study (or at the moment of exposure)? | Selection |
| 7 | Were the outcomes measured in a valid and reliable way? | Measurement |
| 8 | Was the follow up time reported and sufficient to be long enough for outcomes to occur? | Reporting |
| 9 | Was follow up complete, and if not, were the reasons to loss to follow up described and explored? | Attrition |
| 10 | Were strategies to address incomplete follow up utilized? | Reporting |
| 11 | Was appropriate statistical analysis used? | Reporting |

**Studies rating:**

The following table is showing minimum number of stars required in each factor

| Studies rating | Selection | Measurement | Reporting | Attrition |
| --- | --- | --- | --- | --- |
| Good | 2.5 to 3 | 2 | 4 to 5 | 1 |
| Fair | 1.5 to 2 | 1 to 1.5 | 2 to 3.5 | 0.5 |
| Poor | 0 to 1 | 0 to 0.5 | 0 to 1.5 | 0 |

**Star obtained:**

| Selection: |  |  |
| --- | --- | --- |
| Measurement: |  |  |
| Reporting: |  |  |
| Attrition: |  |  |
| **Overall:** |  |  |

**JBI CRITICAL APPRAISAL CHECKLIST FOR COHORT STUDIES**

**Author ___________**Do, et al.,**______________ Year__**2018**_______**

**Assessment and categorization of different factors from JBI scale of longitudinal studies**

| **Item No.** | **Explanation** | **Categorization** |
| --- | --- | --- |
| 1 | Were the two groups similar and recruited from the same population? | 1 |
| 2 | Were the exposures measured similarly to assign people to both exposed and unexposed groups? | 0.5 |
| 3 | Was the exposure measured in a valid and reliable way? | 0.5 |
| 4 | Were confounding factors identified? | 0 |
| 5 | Were strategies to deal with confounding factors stated? | 0 |
| 6 | Were the groups/participants free of the outcome at the start of the study (or at the moment of exposure)? | 0.5 |
| 7 | Were the outcomes measured in a valid and reliable way? | 1 |
| 8 | Was the follow up time reported and sufficient to be long enough for outcomes to occur? | 1 |
| 9 | Was follow up complete, and if not, were the reasons to loss to follow up described and explored? | 1 |
| 10 | Were strategies to address incomplete follow up utilized? | 0 |
| 11 | Was appropriate statistical analysis used? | 1 |

**Studies rating:**

The following table is showing minimum number of stars required in each factor

| Studies rating | Selection | Measurement | Reporting | Attrition |
| --- | --- | --- | --- | --- |
| Good | 2.5 to 3 | 2 | 4 to 5 | 1 |
| Fair | 1.5 to 2 | 1 to 1.5 | 2 to 3.5 | 0.5 |
| Poor | 0 to 1 | 0 to 0.5 | 0 to 1.5 | 0 |

**Star obtained:**

| Selection: | 2 | Fair |
| --- | --- | --- |
| Measurement: | 1.5 | Fair |
| Reporting: | 1.5 | Poor |
| Attrition: | 0.5 | Fair |
| **Overall:** | **5.5** | **Fair** |

**JBI CRITICAL APPRAISAL CHECKLIST FOR COHORT STUDIES**

**Author________**Garenne, et al.,**_____________ Year____**2019**_____**

**Assessment and categorization of different factors from JBI scale of longitudinal studies**

| **Item No.** | **Explanation** | **Categorization** |
| --- | --- | --- |
| 1 | Were the two groups similar and recruited from the same population? | 1 |
| 2 | Were the exposures measured similarly to assign people to both exposed and unexposed groups? | 1 |
| 3 | Was the exposure measured in a valid and reliable way? | 1 |
| 4 | Were confounding factors identified? | 0.5 |
| 5 | Were strategies to deal with confounding factors stated? | 0.5 |
| 6 | Were the groups/participants free of the outcome at the start of the study (or at the moment of exposure)? | 1 |
| 7 | Were the outcomes measured in a valid and reliable way? | 1 |
| 8 | Was the follow up time reported and sufficient to be long enough for outcomes to occur? | 1 |
| 9 | Was follow up complete, and if not, were the reasons to loss to follow up described and explored? | 0.5 |
| 10 | Were strategies to address incomplete follow up utilized? | 0 |
| 11 | Was appropriate statistical analysis used? | 1 |

**Studies rating:**

The following table is showing minimum number of stars required in each factor

| Studies rating | Selection | Measurement | Reporting | Attrition |
| --- | --- | --- | --- | --- |
| Good | 2.5 to 3 | 2 | 4 to 5 | 1 |
| Fair | 1.5 to 2 | 1 to 1.5 | 2 to 3.5 | 0.5 |
| Poor | 0 to 1 | 0 to 0.5 | 0 to 1.5 | 0 |

**Star obtained:**

| Selection: | 3 | Good |
| --- | --- | --- |
| Measurement: | 2 | Good |
| Reporting: | 2 | Fair |
| Attrition: | 0.5 | Fair |
| **Overall:** | **7.5** | **Fair** |
